# PhenotypeSimulator: A comprehensive framework for simulating multi-trait, multi-locus genotype to phenotype relationships

**DOI:** 10.1093/bioinformatics/bty197

**Published:** 2018-03-29

**Authors:** Hannah Verena Meyer, Ewan Birney

**Affiliations:** European Molecular Biology Laboratory, European Bioinformatics Institute (EMBL-EBI), Wellcome Trust Genome Campus, Hinxton, Cambridge, UK

## Abstract

**Motivation:**

Simulation is a critical part of method development and assessment. With the increasing sophistication of multi-trait and multi-locus genetic analysis techniques, it is important that the community has flexible simulation tools to challenge and explore the properties of these methods.

**Results:**

We have developed *PhenotypeSimulator*, a comprehensive phenotype simulation scheme that can model multiple traits with multiple underlying genetic loci as well as complex covariate and observational noise structure. This package has been designed to work with many common genetic tools both for input and output. We describe the underlying components of this simulation tool and illustrate its use on an example dataset.

**Availability and implementation:**

*PhenotypeSimulator* is available as a well documented R/CRAN package and the code is available on github: https://github.com/HannahVMeyer/PhenotypeSimulator.

**Supplementary information:**

Supplementary data are available at *Bioinformatics* online.

## 1 Introduction

The relationship between genotype and phenotype has been a topic for over a 100 years of biological research. There are a multitude of phenotypes for each organism, defined by the observations and measurements one can make on individuals, and these phenotypes can be related to each other in complex ways. Furthermore, the link between genotype and phenotype is equally complex, with many phenotypes being influenced by more than one genetic locus (polygenic effects) and one locus influencing many phenotypes (pleiotropic effects). Finally, a multitude of environmental and measurement effects influence phenotypes. Statistical genetics continues to develop novel ways to analyse genotype to phenotype relationships; models range from simple linear models with genetic variant effects on a single trait to complex linear mixed models (LMMs) with additional genetic and non-genetic random effect components on multiple traits. All models account for observational noise and, usually, known environmental covariates (Loh *et al.*, 2014; Marigorta and Gibson, 2014; Stephens, 2013; Zhou and Stephens, 2014). With the increase in analysis complexity, researchers require sophisticated simulations of realistic genotype and phenotype structures. These simulations are critical for testing methods and exploring the impact of different phenotypic and genetic architectures of biological traits. The simulated genotypes and phenotypes reflect perceived understanding of the true phenotype structure but do not guarantee the biological correctness of real phenotypes. However, they are invaluable in model design, as any model showing flawed statistics on the possibly simplified biological model will suffer from at least the same flaws on the true biological data.

Many simulation packages put a strong focus on the genotype simulation allowing for the simulation of different evolutionary selection processes via forward-time (Carvajal-Rodríguez, 2008; Lambert *et al.*, 2008; Li *et al.*, 2012; Neuenschwander *et al.*, 2008; Peng and Kimmel, 2005) and coalescent-based simulation (Ewing and Hermisson, 2010; Hudson, 2002; Kelleher *et al.*, 2016) frameworks. More recently, re-sampling-based approaches have been developed, where existing genotype data are sampled and combined to generate the genotypes of the simulated samples, while retaining the original allele frequency and LD patterns (Wright *et al.*, 2007; Su *et al.*, 2011). The majority of these methods allow for the simulation of simple case-control phenotypes or a single quantitative phenotype. In contrast, software packages that focus on more complex phenotype simulation often rely on simple genotype simulations (O’Reilly *et al.*, 2012; Porter and O’Reilly, 2017) or only support externally simulated genotype data (Günther *et al.*, 2011; Zhbannikov *et al.*, 2017) (a list of common genotype and phenotype simulation tools and their simulation strategy is given in the Supplementary Material, SupplementaryTable.pdf). Among the software with a focus on phenotype simulation, MultiPhen (O’Reilly *et al.*, 2012) and MultiTraitGWAS (Porter and O’Reilly, 2017) facilitate the simulation of multiple phenotypes per individual. They both offer the simulation of phenotypes with genetic variant effects and an observational noise term with user-defined covariance structure. In addition, MultiTraitGWAS can simulate non-genetic covariate terms.

Here, we introduce *PhenotypeSimulator*, an R/CRAN package for the flexible simulation of phenotypes with different genetic and non-genetic variance components. *PhenotypeSimulator* is a framework focusing on the simulation of phenotypes, with a particular emphasis on complexity of both multiple phenotypes and multiple genetic loci, which is not provided by other multi-phenotype simulation software (O’Reilly *et al.*, 2012; Porter and O’Reilly, 2017). In addition to genetic variant effects, it allows for the simulation of infinitesimal genetic effects (i.e. genetic background) which are a key component of standard LMMs for genetic association studies, various non-genetic covariate effects and noise effects with pre-defined covariance structure. *PhenotypeSimulator* offers similar genotype simulation as used in other software (O’Reilly *et al.*, 2012; Porter and O’Reilly, 2017) and studies (Lippert *et al.*, 2013). We have written *PhenotypeSimulator* to be easily integrated with external genotype simulation software (such as coalescent and forward time simulation and re-sampling approaches) and it can generate output suitable as input for a number of standard genetic association tools such as PLINK (Chang *et al.*, 2015), GEMMA (Zhou and Stephens, 2014) or SNPTEST (Marchini *et al.*, 2007). We demonstrate the usage and application of *PhenotypeSimulator* by simulating phenotypes and using it to evaluate the power of different LMM designs in a genetic association study.

## 2 Materials and methods

### 2.1 Phenotype simulation with *PhenotypeSimulator*

#### 2.1.1 Phenotype components

In *PhenotypeSimulator*, the phenotypes $Y\in R^{N,P}$ of *N* samples and *P* traits are generated as the sum of (i) genetic variant effects $XB\in R^{N,P}$, (ii) infinitesimal genetic effects $U\in R^{N,P}$, (iii) non-genetic covariate effects $WA\in R^{N,P}$, (iv) correlated non-genetic effects $T\in R^{N,P}$ and (v) observational noise effects $\Psi \in R^{N,P}$. For components (i)–(iii) and (v), the user can chose a certain percentage of their variance to be shared across all traits (shared) and the remainder to be independent (ind) across traits. By allowing for a split in variance components, we provide the opportunity to simulate scenarios where the components affect the trait set in a heterogeneous manner, allowing to simulate for instance pleiotropic effects for the genetic variants.

 Genetic variant effects: For the genetic variant effects, *S* random SNPs for *N* samples are drawn from the (simulated) genotypes. From the *S* random SNPs, a proportion θ is selected to be causal across all traits. The shared genetic variant effect is simulated as the matrix product of this shared causal SNP matrix $X^{\text{shared}}\in R^{N,\theta \times S}$ and the shared effect size matrix $B^{\text{shared}}\in R^{\theta \times S,P}$. The columns of the shared effect size matrix are simulated to be perfectly correlated, i.e. the effect of a SNP genetic effect is proportionally the same for all affected traits. The effect sizes for $B^{\text{shared}}$ can either be simulated to have normal or uniform properties. The is implemented as follows in *PhenotypeSimulator*: **B**^shared^ is the matrix product of the two vectors $b_{s}\in R^{\theta \times S,1}$ and $b_{p}^{T}\in R^{1,P}$. To simulate effect sizes with approximately normal properties (Oliveira and Seijas-Macias, 2012; Equations (31)–(33)), *b_s_* and *b_p_* are drawn from two normal distributions, where $\mu_{b_{p}}=0$ and $\sigma_{b_{p}}=1$ and $\mu_{b_{s}}$ and $\sigma_{b_{s}}$ specified by the user. For the simulation of uniformly distributed effect sizes, *b_s_* and $b_{p}^{T}$ are drawn from two exponential distributions whose negative normalized log product yields an approximate uniform distribution (Song, 2005) across the user-defined range. The remaining (1−θ)×*S* SNPs are simulated to have an independent effect across a specified number of traits $P^{\text{ind}}$. To realize this structure, $B^{\text{ind}}\in R^{(1-\theta )\times S,P}$ is initialized with either normally or uniformly distributed entries, with *μ_B_* and *σ_B_* as specified by the user (same as for shared effect). Subsequently, $P-P^{\text{ind}}$ traits are randomly selected and the row entries for **B**^ind^ at these traits set to zero. The independent genetic variant effect is the matrix product of $X^{\text{ind}}\in R^{N,(1-\theta )\times S}$ and **B**^ind.^

Non-genetic covariate effects: The non-genetic covariate effects are based on *K* non-genetic covariates $W\in R^{N,K}$, with a proportion *γ* being shared across all traits yielding the shared covariates matrix $W^{\text{shared}}\in R^{N,\gamma \times K}$. The proportion of 1−γ non-genetic covariates that are independent make up the independent covariates matrix $W^{\text{ind}}\in R^{N,(1-\gamma )\times K}$. The distributions for each of the *K* non-genetic covariates are independent and can be either normal, uniform, binomial or categorical. The distribution and respective parameters are chosen by the user. The effect size matrices $A^{\text{shared}}\in R^{\gamma \times K,P}$ and $A^{\text{ind}}\in R^{(1-\gamma )\times K,P}$ were designed as described for the genetic effects. The final non-genetic covariate effects are the matrix product of the covariate matrices and their effect size matrices: $W^{\text{ind}}A^{\text{ind}}$ and $W^{\text{shared}}A^{\text{shared}}$.

Infinitesimal genetic effects: The basis of the infinitesimal genetic effect **U** is the *N *×* N* genetic relationship matrix **K**, either estimated from the genotypes of the simulated samples as $\frac{1}{m}XX^{T}$, where *m* is the mean value of the diagonal elements of $XX^{T}$ or provided by the user. A suitable model for simulating the infinitesimal genetic effect $U\in R^{N,P}$ with the known *N *×* N* sample covariance **K** and trait covariance **C** is a multivariate normal distribution (as for instance by Casale *et al.*, 2015; Zhou and Stephens, 2014) where

(1) $$\text{vec}(U)∼N_{N\times P}\left(\text{vec}(0),C⊗K\right).$$

The structure of **C** depends on the desired design of the covariance effect, which can be either shared or independent across traits. This distribution can be realized by simulating a random variable $Z\in R^{M,L}$ as iid N(0,1) and setting

(2) $$\text{vec}(U)=BZA^{T}$$

where $B\in R^{N,M}$ reflects the genetic relationship i.e. sample covariance with $K=BB^{T}$ and $A\in R^{P,L}$ the trait covariance with $C=AA^{T}$, respectively (*M* and *L* depend on the rank of *K* and *C*, hence are bound by *N* and *P*). A detailed derivation of Equation (2) from Equation (1) can be found in the Supplementary Material (SimulationSchemeInfinitesimalGeneticEffect.pdf) and has similarly been applied in (Casale *et al.*, 2015). By recasting Equation (1) as Equation (2), the infinitesimal genetic effect **U** described by a multivariate normal distribution is effectively modelled as the product of three matrices, representing the sample covariance (**B**), a normally distributed variable (**Z**) and the trait covariance (**A**). Different designs of **A** will allow for the simulation of shared and independent genetic random effects. For the independent effect, **A**^ind^ is a diagonal matrix with normally distributed entries: ${\left(A^{\text{ind}}\right)}^{T}=\text{diag}(a_{1},a_{2},\ldots ,a_{P})∼N\left(0,1\right)$, such that $U^{\text{ind}}=\text{vec}(BZ{\left(A^{\text{ind}}\right)}^{T})$. **A**^shared^ of the shared effect is simulated as a matrix of column rank one, with normally distributed entries in column one and zeros elsewhere: $a_{i,1}∼N\left(0,1\right)$ and $a_{i,j\neq 1}=0$ such that $U^{\text{shared}}=\text{vec}(BZ{\left(A^{\text{shared}}\right)}^{T})$.

Correlated non-genetic effects: Correlated non-genetic effects are simulated as a multivariate normal distribution with a covariance matrix described by a defined trait-by-trait correlation. Any correlation structure between the phenotypes can be simulated with this effect component, as the desired correlation matrix **C** can be supplied by the user. In addition, as a simple approximation for spatially correlated phenotypes (as they might occur, for instance, in image-based phenotypes, for an example, see Supplementary Material, SimulationBasedOnExampleData.pdf), *PhenotypeSimulator* provides the construction of such a **C** as follows: traits of distance *d* = 1 (adjacent trait columns) will have the highest specified correlation *r*, traits with *d* = 2 have a correlation of *r*^2^, up to traits with d=(P−1) with a correlation of $r^{\left(P-1\right)})$, such that the correlation is highest at the first off-diagonal element and decreases exponentially by distance from the diagonal. The correlated non-genetic effect matrix is simulated as $T∼N_{N\times P}\left(0,C\right)$.

Observational noise: The observational noise effects Ψ are simulated as the sum of a shared and an independent observational noise effect. Both effect components are simulated by the matrix product of $B\in R^{N,P}∼N\left(0,1\right)$ with $A\in R^{P,P}$. To realize the shared effect $\Psi^{\text{shared}}$ (which introduces perfect correlation between the traits in this component), **A**^shared^ is simulated as a matrix of row rank one, with normally distributed entries in row one and zeros elsewhere: $a_{1,j}∼N\left(0,1\right)$ and $a_{i\neq 1,j}=0$. **A** of the independent component is a diagonal matrix with normally distributed entries: ${\left(A^{\text{ind}}\right)}^{T}=\text{diag}(a_{1},a_{2},\ldots ,a_{P})∼N\left(0,1\right)$.

*PhenotypeSimulator* requires at least one phenotype component to simulate the phenotypes. Components can be combined as specified by the user and the correlation they introduce in the trait structure can be controlled by the specified levels of independent and shared effects (at the extremes, components can be simulated to either only have shared or independent effects). If desired, a simple phenotype structure following a model as cast, for instance, in the multivariate normal model by Zhou and Stephens (2014) can be achieved by specifying only genetic variant effects, non-genetic covariate effects, infinitesimal genetic effects and observational noise.

#### 2.1.2 Scaling and phenotype construction

*PhenotypeSimulator* enables the specification of the amount of variance that each component should contribute to the total phenotypic variance. Every component is thereby scaled by a factor *a* such that its average column variance ${\bar{V}}_{col}=\frac{V_{1}+\cdots +V_{p}}{p}$ explains a specified percentage *x* of the total variance:

(3) $$a=\sqrt{x{\bar{V}}_{\text{col}}{}^{-1}}$$

The final simulated phenotype **Y** is expressed as the sum of the scaled genetic variant effects, the non-genetic covariates, the correlated non-genetic effects and observational noise effects:

(4) $$\begin{array}{cc} Y & =X^{\text{shared}}B^{\text{shared}}+X^{\text{ind}}B^{\text{ind}}+W^{\text{shared}}A^{\text{shared}}+W^{\text{ind}}A^{\text{ind}}\\ & +U^{\text{shared}}+U^{\text{ind}}+T+\Psi^{\text{shared}}+\Psi^{\text{ind}}. \end{array}$$

### 2.2 Case study

The analysis code and parameters of this case study, from the data simulation to the genome-wide association study, can be found in the Supplementary Material (Simulation-and-LinearModel.pdf).

#### 2.2.1 Data simulation

Genotypes (8 073 414 genetic variants) for 1000 individuals were simulated via Hapgen2 (Su *et al.*, 2011) (re-sampling approach), based on the European Samples of the 1000 Genomes project (1000 Genomes Project Consortium, 2012). Phenotypes were simulated with *PhenotypeSimulator*, using the simulated genotypes as basis for the SNP and infinitesimal genetic effects. A total of three phenotypes for 1000 samples with the ten SNP genetic effects shared across all traits (randomly sampled from the simulated genotypes), four non-genetic covariates, an infinitesimal genetic, a correlated noise and an observational noise effect were simulated. For the genetic variant effects, only shared effects across traits were simulated. For the remainder of the phenotype component, 80% of their variance was simulated to be shared across all traits while the remaining proportion of variance remained independent. The total genetic variance was set to 40%, leaving 60% of variance explained by the noise terms.

#### 2.2.2 Genome-wide association study

The simulated genotypes, phenotypes, kinship and covariates were used in GWAS. Two different types of GWAS were conducted (i) a multi-trait association study, jointly mapping all three traits and (ii) single-trait association studies, where each trait was individually tested for association with the genotypes. Single-trait GWAS was run for all three traits. All GWAS were conducted with GEMMA (version 0.96) (Zhou and Stephens, 2014). In both, the multi-trait and single-trait GWAS, the phenotypes (-p flag) were modeled as the sum of genetic (simulated SNPs; -g flag) and non-genetic (simulated covariates; -c flag) fixed effects, a random genetic effect (with the eigenvectors and values of the kinship matrix, -u and -d flag) and observational noise (LMM with likelihood ratio test using the -lmm 2 flag). For a comparison of the number of causal SNPs recovered in the multi-trait and single-trait GWAS, the *P*-values of the single-trait GWAS were adjusted by the number of test conducted (Bonferroni adjustment for three tests).

## 3 Results

**Fig. 1.**
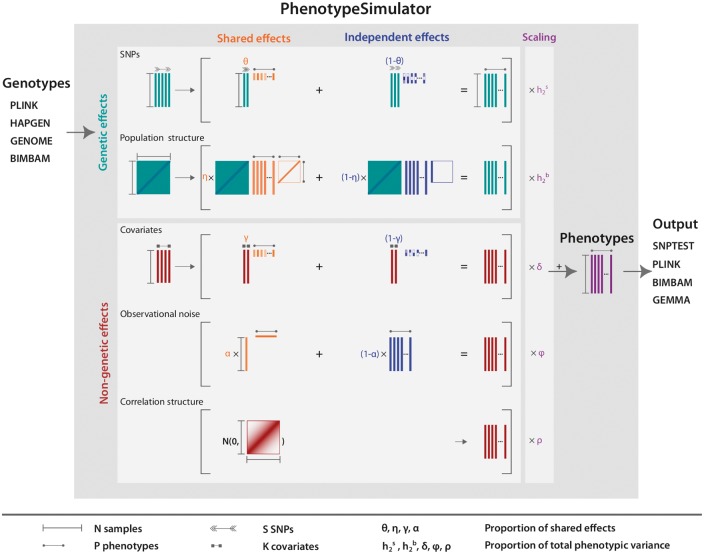
Phenotype simulation scheme. *PhenotypeSimulator* takes genotypes from a number of different input formats and uses these as the basis for the simulation of the genetic effects. In addition to the genetic effects, non-genetic covariates, observational noise and non-genetic correlation structure can be simulated. The effect structure of the upper four components can be divided into a shared effect across traits or an independent effect for a number of traits, allowing for complex phenotype structures such as the simulation of pleiotropy. Before combining the phenotype components, they are scaled to a user-defined proportion of the total phenotypic variance. Finally, the simulated phenotype and its components can be saved into a number of different genetic output formats. Arrows, lines and rectangles mark the dimensions of each component

*PhenotypeSimulator* works by:

1. Simulating or importing genotypes (Fig. 1, genotypes),
2. simulating genetic and non-genetic phenotype components of interest (Fig. 1, light grey boxes),
3. scaling each component according to a certain proportion of variance explained (Fig. 1, scaling),
4. combining re-scaled phenotype components into a final phenotype (Fig. 1, phenotypes), and
5. saving phenotypes and genotypes in standard output formats (Fig. 1, output).

*PhenotypeSimulator* can simulate simple bi-allelic SNPs, where each SNP is simulated from a binomial distribution with two trials and probability equal to the given allele frequencies [as for instance used in (Lippert *et al.*, 2013)]. This simple approach, however, does not simulate any dependency between the genotypes as is observed with LD structure in the genome. To allow for more complex genotype structures, *PhenotypeSimulator* can import genotypes generated from different genotype simulation software, covering genotypes simulated from coalescent models GENOME (Liang *et al.*, 2007), a re-sampling-based approach HAPGEN2 (Su *et al.*, 2011) and different forward-time approaches delimited-formats as given by simuPOP (Peng and Kimmel, 2005), ForSim (Lambert *et al.*, 2008) and GenomePop2 (Carvajal-Rodríguez, 2008). In addition, standard genotype formats such as PLINK (Chang *et al.*, 2015) or BIMBAM (Guan and Stephens, 2008) are supported.

These genotypes form the basis for the simulation of the genetic components of the phenotypes: genetic variants that are associated with the phenotype and infinitesimal genetic effects simulating underlying population structure and relatedness in a cohort. In addition, *PhenotypeSimulator* offers the simulation of other non-genetic components, which reflect environmental, experimental or other unexplained variance in the data. Although in many genetic association studies, the sources of non-genetic correlation are often combined, we have found it valuable to separate these components to explore the impact of different correlation structures from these sources. The environmental components can be simulated as covariates with different distributions mimicking influences such as sex (binary), age (uniform/normal) or country of origin (categorical), correlated non-genetic effects and observational noise. Correlated non-genetic effects can be used to simulate a phenotype component with a defined level of correlation between traits. For instance, such effects can reflect correlation structure decreasing in phenotypes with ordered or spatial components, e.g. in imaging data. Observational noise captures any non-specified effects that arise due to, e.g. experimental measurement error. *PhenotypeSimulator* can also be used with a combined non-genetic covariance model, similar to more standard LMMs (O’Reilly *et al.*, 2012; Porter and O’Reilly, 2017; Zhou and Stephens, 2014).

The proportion of variance assigned to each component will differ depending on the biological understanding of the simulated phenotype. *PhenotypeSimulator* allows for the specification of these variance proportions and, in addition, provides the option to divide the explained variance into two components: one that is shared across phenotypes and a second component that acts independently on certain phenotypes. For instance, the level of shared and independent effects for a genetic variant allows for the simulation of different levels of pleiotropy.

There are many ways to simulate these phenotype components depending on the scope and the model to be tested. Typically, it is assumed that the overall phenotype structure is well represented by an additive linear combination of individual components (Loh *et al.*, 2014; Marigorta and Gibson, 2014; Stephens, 2013; Zhou and Stephens, 2014). *PhenotypeSimulator* assumes this phenotype structure and sums the individual phenotype components to generate the final phenotypes.

The simulated genotypes and phenotypes can automatically be written into a number of formats for standard genetic association software such as PLINK (Chang *et al.*, 2015), BIMBAM (Guan and Stephens, 2008), GEMMA (Zhou and Stephens, 2014) or SNPTEST (Marchini *et al.*, 2007).

**Fig. 2.**
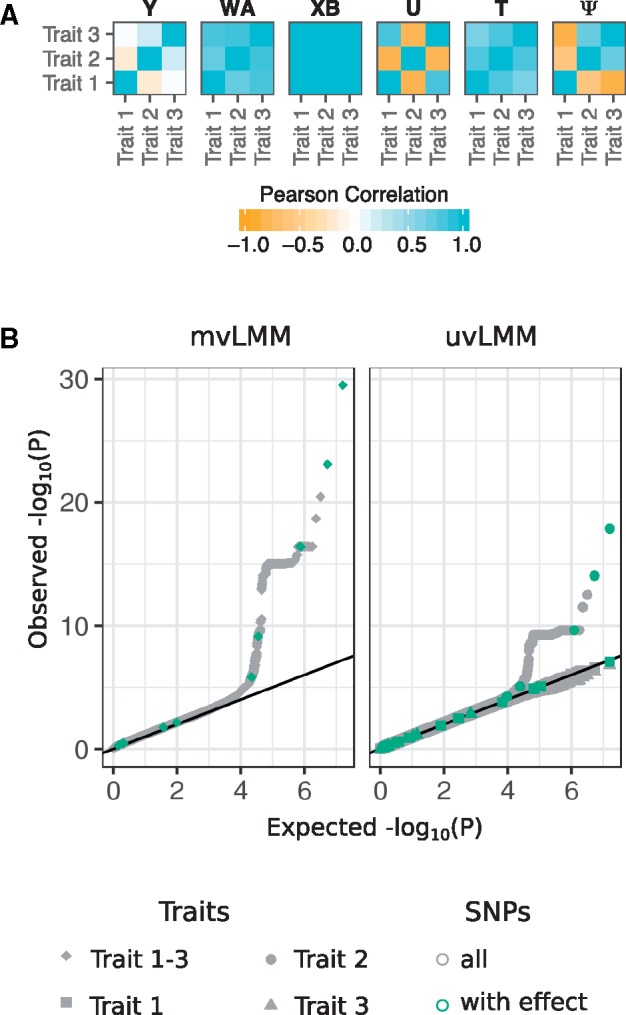
Phenotype simulation and genome-wide association study as a downstream application. (**A**) Heatmaps of the trait-by-trait correlation (Pearson correlation) of a simulated phenotype **Y** and its five phenotype components: genetic variant effects **XB**, infinitesimal genetic effects **U**, non-genetic covariates **WA**, correlated non-genetic effects **T** and observational noise Ψ. The non-genetic covariates consist of four independent components, two following a binomial and two following a normal distribution. The genetic variant effect of ten causal SNPs with shared effect across all traits, yielding the strong correlation structure observed above (see Section 2). (**B**) Quantile-quantile plots of *P*-values observed from a multivariate linear mixed model (mvLMM) and univariate linear mixed models (uvLMM) fitted to each of the about eight million genome-wide SNPs (grey), including the ten SNPs for which a phenotype effect was modelled (green). The R code and detailed description of the simulation and analysis are provided in the Supplementary Material (Simulation-and-LinearModel.pdf)

To demonstrate the usage and application of *Phenotype Simulator*, we simulated a set of phenotypes and used them to evaluate the power of different LMM designs in genome-wide association studies (GWAS). We simulated genotype data for 1000 individuals via a re-sampling-based approach (Su *et al.*, 2011), mimicking population structure from four populations in the 1000 Genomes project (1000 Genomes Project Consortium, 2012). We generated a phenotype set consisting of 3 traits with 10 genetic variant effects and 4 non-genetic covariates. For 10 genetic variant effects, we randomly selected 10 variants from the genotypes and simulated shared genetic variant effects across all phenotypes. We introduced additional correlation structure by including an infinitesimal genetic effect based on the individuals’ kinship estimates as well as a non-genetic correlated and an observational noise effect (parameters and R code in the Supplementary Material, Simulation-and-LinearModel.pdf). Figure 2A shows the trait-to-trait correlations of the final phenotype and each of its components. The final phenotypes served as the response variable in the GWAS based on LMM with the simulated SNPs and non-genetic covariates as fixed effects and the kinship estimated from the genotypes as part of the genetic random effect (Zhou and Stephens, 2014). We analysed the power of jointly modeling all three phenotypes (multivariate LMM) and the power of univariate models where the association of each phenotype is analysed separately (Fig. 2B). For our simulated phenotypes with shared genetic variant effects only, the multi-trait GWAS shows a greater power compared to any of the single trait analyses. The multi-trait GWAS detected 4 out of 10 SNPs for which a phenotype effect was modelled that pass the commonly used genome-wide significant threshold of 5 × 10^−8^ (Fadista *et al.*, 2016). The single-trait GWAS only recovered three of these SNPs. The ability of linear (mixed) models to detect the SNPs for which a phenotype effect was modeled depends on the allele frequencies of these SNPs and the effect size (Cohen, 1992; Halsey *et al.*, 2015): the higher the effect size and/or the allele frequencies the better the power to detect the SNP effects. The *P*-values of all SNPs with simulated effect on the phenotypes in relation to their allele frequencies and simulated effect sizes can be found in the Supplementary Material (Simulation-and-LinearModel.pdf), showing a strong trend for SNPs with high allele frequencies and large simulated effect sizes to have low *P*-values.

## 4 Conclusion

*PhenotypeSimulator* offers a framework for complex multi-trait, multi-locus phenotype simulations in quantitative genetics packaged in an easy to use manner for statistical geneticists. There are a variety of key features of *PhenotypeSimulator* that have both driven its development and usage. First, it is the only simulation package that we know that can simulate complex multi-trait phenotypes with complex multi-locus genetics, including a population structure term with phenotypic correlation. Second, realistic covariate structures can be created with similar properties (e.g. categorical covariates or covariates drawn from different distributions) to real covariates. Third, the different components can be independently extracted and scaled, for example having the ‘true’ variance components and covariance matrices from the simulation readily available for comparison to inference schemes. Finally, we have developed *PhenotypeSimulator* as a flexible component in the standard genetics pipeline, with the ability to both read genetic formats from well used tools and output phenotypes compatible with many tools. This allows easy large-scale deployment for comprehensive simulation across many parameter settings. The underlying model for *PhenotypeSimulator* corresponds to the common place LMM framework. As such, it is limited in its use for benchmarking between methods, where LMMs methods are likely to perform best. However, the need for an underlying model is true for any simulation package. We have extensively documented *PhenotypeSimulator* for ease of use, providing vignettes for sample genotype simulation with the supported genotype simulation tools (Supplementary Material, sample-scripts-external-genotype-simulation.pdf), a user manual for *PhenotypeSimulator* (Supplementary Material, UsagePhenotypeSimulator.pdf) and a full application documentation from genotype simulation to GWAS (Supplementary Material, Simulation-and-LinearModel.pdf). Furthermore, the code is present on github (https://github.com/HannahVMeyer/PhenotypeSimulator) and we welcome other additions to this tool. For example, although we currently model polygenic and pleiotropic effects, we have not yet modelled epistatic effects and would enthusiastically accept extensions in this area.

## Supplementary Material

Supplementary Data
