# Supplementary material for: PhenotypeSimulator: A comprehensive framework for simulating multi-trait, multi-locus genotype to phenotype relationships
Source: Bioinformatics. 2018 Mar 29;34(17):2951–6. doi: 10.1093/bioinformatics/bty197 (PMC6129313; doi:10.1093/bioinformatics/bty197)
Supplement: Supplementary Data [file bty197_supp.zip › bty197-suppl_data/Sample-scripts-external-genotype-simulation.pdf]

Genotype simulation supported by *PhenotypeSimulator*

Hannah Meyer

2018-02-23

There are a number of different strategies to generate genotype data for genetic association studies.

1. **Simple bi-allelic SNPs without structure**: In the most simple case and assuming bi-allelic SNPs, each SNP is simulated from a binomial distribution with two trials and probability equal to the given allele frequencies. This simple approach does not simulate any dependency between the genotypes as is observed with LD structure in the genome. Simple bi-allelic SNPs can be simulated with PhenotypeSimulator::simulateGenotypes or via PLINK [1]. For large datasets (~ 1 million SNPs), simulation via PLINK is recommended.
2. **Coalescent approaches**: Coalescent methods simulate genealogical events backward in time. These events typically include the coalescence of two sequences into a single ancestral lineage, recombination within a sequence or migration between populations. A coalescent-based approach to simulate whole genome data is implemented in GENOME [2] and its output format is supported as an input format for *PhenotypeSimulator*.
3. **Forward-time approaches**: Forward-time simulation methods evolve a population forward in time, subject to arbitrary genetic and demographic factors [3]. Several algorithms are available, many of which allow customisation by building the simulation scheme in R or python, hence the output format of the genotypes can be specified by the user. *PhenotypeSimulator* offers reading genotypes from delimited-files and as such, any genotypes generated by these programmes can be read (e.g. MetaSim [4] or simuPop [5]).
4. **Resampling approaches**: Resampling-based approaches combine existing genotype data into the genotypes of the simulated samples, thereby retaining allele frequency and LD patterns [6]. A standard resampling-based approach that uses common genotype formats (common for the oxford genetics format) is Hapgen2 and an example usage is given below.

Examples for genotype simulation via PLINK, GENOME and Hapgen2 are given below. The corresponding data can be found in the extdata folder.

Simple bi-allelic SNPs without structure via PLINK

Download PLINK and create a folder for the PLINK output files. The example below uses PLINK to simulate 1000 SNPs, with allele frequencies between 0 and 1 for 100 controls and 0 cases. PhenotypeSimulator::readStandardGenotypes reads the resulting .bim, .fam, .bed files.

```
# Serves as output directory for simulation and parameter file
mkdir -p ~/PhenotypeSimulator/inst/extdata/genotypes/plink
cd ~/PhenotypeSimulator/inst/extdata/genotypes/plink

# Write paramter file to simulate 1000 SNPs, with allele frequencies between 0
# and 1 for 100 controls and 0 cases
echo -e "1000\tSNP\t0.00\t1.00\t1.00\t1.00" > plink_sim_par.txt

plink --simulate plink_sim_par.txt \
      --simulate-ncases 0 \
```

```
      --simulate-ncontrols 100 \
      --out genotypes_plink
```

Coalescent simulation via Genome

Download GENOME and create a folder for the GENOME output files. The example below uses GENOME to simulate genetic data for a population comprised of three sub-populations with 30, 30 and 40 samples. The simulated POPULATION PROFILE, SNP positions and the genotypes are all saved in genotypes_genome.txt. PhenotypeSimulator::readStandardGenotypes reads genotype information by parsing this output file and extracting the samples information following the line 'Samples:'

```
mkdir -p ~/PhenotypeSimulator/inst/extdata/genotypes/genome
cd ~/PhenotypeSimulator/inst/extdata/genotypes/genome

#  subpopulation with 30, 30 and 40 individuals each
genome -pop 3 30 30 40 > genotypes_genome.txt
```

Resampling-based simulation via Hapgen2

Download hapgen2, hapgen example files and the 1000Genomes data from the impute2 webpage as starting point for the resampling-based genotype simulation with hapgen2. The example files contain example haplotypes (ex.haps), legend files (ex.leg) map (ex.map) and TagSNP files (ex.tags). The following examples simulates genotype data for 100 controls and 0 cases with 1000 SNPs from chromosome 1. PhenotypeSimulator::readStandardGenotypes reads the resulting .gen and .samples files.

```
# contains ex.leg file and serves as output directory
mkdir -p ~/PhenotypeSimulator/inst/extdata/genotypes/hapgen
cd ~/PhenotypeSimulator/inst/extdata/genotypes/hapgen

# contains 1000Genomes haplotype data used for sampling
hapdir=/path/to/CEU.0908.impute.files

hapgen2 -m $hapdir/genetic_map_chr1_combined_b36.txt \
        -l ex.leg \
        -h $hapdir/CEU.0908.chr1.hap \
        -o genotypes_hapgen \
        -n 100 0 \
        -dl 45162 0 0 0 \
        -no_haps_output
```

References

1. Chang CC, Chow CC, Tellier LC, Vattikuti S, Purcell SM, Lee JJ. Second-generation PLINK: rising to the challenge of larger and richer datasets. GigaScience. 2015;4: 7. doi:10.1186/s13742-015-0047-8

2. Liang L, Zollner S, Abecasis GR. GENOME: a rapid coalescent-based whole genome simulator. Bioinformatics. Oxford University Press; 2007;23: 1565–1567. doi:10.1093/bioinformatics/btm138

3. Peng B, Amos CI. Forward-time simulation of realistic samples for genome-wide association studies. BMC Bioinformatics. BioMed Central; 2010;11: 442. doi:10.1186/1471-2105-11-442

4. Strand AE. Metasim 1.0: An individual-based environment for simulating population genetics of complex population dynamics. Molecular Ecology Notes. Blackwell Publishing, Ltd; 2002;2: 373–376.

doi:10.1046/j.1471-8286.2002.00208.x

5. Peng B, Kimmel M. simuPOP: a forward-time population genetics simulation environment. Bioinformatics. Oxford University Press; 2005;21: 3686–3687. doi:10.1093/bioinformatics/bti584

6. Wright FA, Huang H, Guan X, Gamiel K, Jeffries C, Barry WT, et al. Simulating association studies: a data-based resampling method for candidate regions or whole genome scans. Bioinformatics. Oxford University Press; 2007;23: 2581–2588. doi:10.1093/bioinformatics/btm386
