# Supplementary material for: PhenotypeSimulator: A comprehensive framework for simulating multi-trait, multi-locus genotype to phenotype relationships
Source: Bioinformatics. 2018 Mar 29;34(17):2951–6. doi: 10.1093/bioinformatics/bty197 (PMC6129313; doi:10.1093/bioinformatics/bty197)
Supplement: Supplementary Data [file bty197_supp.zip › bty197-suppl_data/Simulation-and-LinearModel.pdf]

Application of PhenotypeSimulator for a power analysis study

Hannah Meyer

2018-03-01

Contents

Genotype simulation via Hapgen2 . . . 1
Phenotype simulation via PhenotypeSimulator . . . 3
Genome-wide association study via GEMMA . . . 6
References . . . 14

This vignette demonstrates the usage and application of *PhenotypeSimulator*. It illustrates the workflow for generating a genetic and phenotypic dataset used for a simple model comparison. First, it shows an example of how to simulate genome-wide SNPs from a re-sampling based approach (Hapgen2). The genotypes can then be used with *PhenotypeSimulator* to generate multiple correlated phenotypes with defined genetic and non-genetic fixed effects as well as correlation induced by kinship structure. The simulated phenotypes, genotypes, non-genetic covariates and kinship are then used with a linear mixed model framework (GEMMA) to investigate the power of multivariate (all phenotypes are analysed jointly) and univariate models (each phenotype is analysed separately) for genome-wide association studies (GWAS).

Chunks that demonstrate the usage of external software in bash (Hapggen2 and GEMMA) and chunks that depend on the fully simulated genotypes or GWAS results are simply echoed and not evaluated. Chunks that show summary statistics and correlation plots are evaluated when building this vignette and use the summary data provided in the `extdata` folder of the package (the .Rmd version of the vignette shows the chunks saving the summary data).

Genotype simulation via Hapgen2

Hapgen2 [1] is a resampling-based genotype simulation tool. As such it needs a reference data set from which to sample genotypes. In the following, the 1000Genomes data from the impute2 webpage was used as the reference dataset. Hapgen2 and its user manual can be found and downloaded from here.

The following code chunk simulates genotypes for 1000 control individuals (no cases) on chromosomes chr1 to chr22. Subsequently, the number of variants on chr5, chr7 and chr11 are counted. These chromosomes were arbitrarily chosen to contain the causal variants. We will use those numbers later as input for *PhenotypeSimulator* (providing the number of variants on the causal chromosomes is optional, but will speed up the simulation procedure).

```
hapdir=~/data/hmeyer/Supplementary/CEU.0908.impute.files

for chr in `seq 1 22`; do
    # Can't get hapgen to work witout the -dl flag (should be possible since
    #v2.0.2) so create dummy variable, that can be passed on
    dummyDL=`sed -n '2'p $hapdir/CEU.0908.chr${chr}.legend | cut -d ' '  -f 2`

    hapgen2 -m $hapdir/genetic_map_chr${chr}_combined_b36.txt \
            -l $hapdir/CEU.0908.chr${chr}.legend \
            -h $hapdir/CEU.0908.chr${chr}.hap \
            -o $hapdir/genotypes_chr${chr}_hapgen \
            -n 1000 0 \
```

```
            -dl $dummyDL 0 0 0 \
            -no_haps_output
done

chr5=`wc -l $hapdir/genotypes_chr5_hapgen.controls.gen |cut -d " " -f 1`
chr7=`wc -l $hapdir/genotypes_chr7_hapgen.controls.gen |cut -d " " -f 1`
chr11=`wc -l $hapdir/genotypes_chr11_hapgen.controls.gen |cut -d " " -f 1`
```

For the estimation of the kinship between the individuals we make use of PLINK [2]. By specifying –oxford-single-chr, we indicate that the input format is the 'oxford' format (.gen/.sample format, see here) and convert the data into plink format (–make-bed). We then strictly prune the genotypes (–indep-pairwise and –extract) and save the chromosome-wide output file names into a file. This file is used for merging the chromosome-wide files into a single, genome-wide genotype file. Finally, we use this pruned, genome-wide SNP file to estimate the genetic relationship of the samples.

```
cd $hapdir

# convert to plink format and prune SNPs
for chr in `seq 1 22`; do
    plink --data genotypes_chr${chr}_hapgen.controls \
        --oxford-single-chr $chr \
        --make-bed \
        --out  genotypes_chr${chr}_hapgen.controls

    plink --bfile genotypes_chr${chr}_hapgen.controls \
        --indep-pairwise 50kb 5 1 \
        --out genotypes_chr${chr}_hapgen.controls

    plink --bfile genotypes_chr${chr}_hapgen.controls  \
        --extract genotypes_chr${chr}_hapgen.controls.prune.in \
        --make-bed \
        --out genotypes_chr${chr}_hapgen.controls.pruned


    echo -e "genotypes_chr${chr}_hapgen.controls.pruned" >> file_list
done

# Merge chromsome-wide files into a single, genome-wide file
plink --merge-list file_list --make-bed --out genotypes_genome_hapgen.controls

for chr in `seq 1 22`; do
    plink --bfile genotypes_chr${chr}_hapgen.controls.pruned \
          --exclude genotypes_genome_hapgen.controls-merge.missnp \
          --make-bed \
          --out genotypes_chr${chr}_hapgen.controls.nodup
    echo -e "genotypes_chr${chr}_hapgen.controls.nodup" >> file_list_nodup
done

plink --merge-list file_list_nodup --allow-no-sex \
      --make-bed \
      --out genotypes_genome_hapgen.controls

# compute kinship
plink --bfile genotypes_genome_hapgen.controls\
```

```
        --make-rel square \
        --out genotypes_genome_hapgen.controls.grm
```

```r
# load kinship data, add small value to diagonal for numerical stability and
# do an eigendecomposition
indir <- "~/data/hmeyer/Supplementary/CEU.0908.impute.files"

kinship <- read.table(paste(indir,
                            "/genotypes_genome_hapgen.controls.grm.rel",sep=""),
                      sep="\t", header=FALSE)

kinship <- as.matrix(kinship)
diag(kinship) <- diag(kinship) + 1e-4

kinship_decomposed <- eigen(kinship)
write.table(kinship,
            paste(indir, "/genotypes_genome_hapgen.controls.grm.rel",
                  sep=""),
            sep="\t", col.names=FALSE, row.names=FALSE)

write.table(kinship_decomposed$values,
            paste(indir, "/genotypes_eigenvalues",
                  sep=""),
            sep="\t", col.names=FALSE, row.names=FALSE)

write.table(kinship_decomposed$vectors,
            paste(indir, "/genotypes_eigenvectors",
                  sep=""),
            sep="\t", col.names=FALSE, row.names=FALSE)
```

Phenotype simulation via PhenotypeSimulator

We simulate a phenotype set consisting of three traits for 1000 samples with ten genetic variant effects shared across all traits (`theta=1`; SNPs randomly sampled from the simulated genotypes) and four non-genetic covariate effects. Additional correlation between the phenotypes is simulated, induced by i) the genetic structure of the individuals (infinitesimal genetic effect through estimated kinship), ii) a correleated non-genetic effect (i.e. correlated measurement noise) and iii) an observational noise effect. For each effect component its proportion of the total genetic variance is specified (the NrSNPsChromsome in the `runSimulation` function are the values we obtained from "chr5=`wc -l $hapdir/genotypes_chr5_hapgen.controls.gen |cut -d " " -f 1`" etc. above). The non-genetic covariates consist of four independent components, two following a binomial and two following a normal distribution. The genetic variant effect was simulated to only show shared effects across traits. The remainder of the components were simulated with 80% of their variance shared across all traits, while the rest remained independent. The total genetic variance accounted to 40% leaving 60% of variance explained by the noise terms.

```r
indir <- "/homes/hannah/data/hmeyer/Supplementary/CEU.0908.impute.files"

# specify directory to save data; if it doesn't exist yet, create i.e. needs
# writing permissions
datadir <- '/homes/hannah/tmp/phenotypeSimulator'
if (!dir.exists(datadir)) dir.create(datadir, recursive=TRUE)

# specify filenames and parameters
```

```r
totalGeneticVar <- 0.4
totalSNPeffect <- 0.1
h2s <- totalSNPeffect/totalGeneticVar
kinshipfile <- paste(indir, "/genotypes_genome_hapgen.controls.grm.rel",
                                sep="")

genoFilePrefix <- paste(indir, "/genotypes_", sep="")
genoFileSuffix <- "_hapgen.controls.gen"

# simulate phenotype with three phenotype components
simulation <- runSimulation(N = 1000, P = 3, cNrSNP=10, seed=43,
                           kinshipfile = kinshipfile,
                           oxgen = TRUE,
                           genoFilePrefix = genoFilePrefix,
                           genoFileSuffix = genoFileSuffix,
                           chr = c(5,7,11),
                           mBetaGenetic = 0, sdBetaGenetic = 0.2,
                           theta=1,
                           NrSNPsOnChromosome=c(533966, 503129, 428863),
                           genVar = totalGeneticVar, h2s = h2s,
                           phi = 0.6, delta = 0.2, rho=0.2,
                           NrFixedEffects = 2, NrConfounders = c(2, 2),
                           distConfounders = c("bin", "norm"),
                           probConfounders = 0.2,
                           genoDelimiter=" ",
                           kinshipDelimiter="\t",
                           kinshipHeader=FALSE,
                           verbose = TRUE )
```

Figure 1 shows heatmaps of the trait-trait correlation (Pearson correlation) of the final phenotype $\mathbf{Y}$ and its five phenotype components: genetic variant effects $\mathbf{XB}$, non-genetic covariate effects $\mathbf{WA}$, relatedness effects $\mathbf{U}$ (infinitesimal genetic effects), non-genetic correlated effects $\mathbf{T}$ and observational noise $\mathbf{\Psi}$.

```r
cor_phenotype <- reshape2::melt(cor(simulation$phenoComponentsFinal$Y))
cor_phenotype$type <- "Y"

cor_genBg <- reshape2::melt(cor(simulation$phenoComponentsFinal$Y_genBg))
cor_genBg$type <- "U"

cor_genFixed <- reshape2::melt(cor(simulation$phenoComponentsFinal$Y_genFixed))
cor_genFixed$type <- "XB"

cor_noiseFixed <- reshape2::melt(cor(simulation$phenoComponentsFinal$Y_noiseFixed))
cor_noiseFixed$type <- "WA"

cor_noiseBg <- reshape2::melt(cor(simulation$phenoComponentsFinal$Y_noiseBg))
cor_noiseBg$type <- "Psi"

cor_correlatedBg <- reshape2::melt(cor(simulation$phenoComponentsFinal$Y_correlatedBg))
cor_correlatedBg$type <- "T"

cor_components <- rbind(cor_phenotype, cor_genBg, cor_genFixed, cor_noiseBg,
                        cor_noiseFixed, cor_correlatedBg)
cor_components$type <- factor(cor_components$type, levels=c("Y",
```

```
                                                                         "WA",
                                                                         "XB",
                                                                         "U",
                                                                         "T",
                                                                         "Psi"),
                                                                 labels=c("bold(Y)",
                                                                         "bold(WA)",
                                                                         "bold(XB)",
                                                                         "bold(U)",
                                                                         "bold(T)",
                                                                         "bold(Psi)"))
colnames(cor_components) <- c("TraitA", "TraitB", "Correlation", "type")

# For prettier plotting
cor_components$TraitA <- gsub("_", " ", cor_components$TraitA)
cor_components$TraitB <- gsub("_", " ", cor_components$TraitB)

p_corr <- ggplot(data=cor_components, aes(x=TraitA, y=TraitB, fill=Correlation))
p_corr <- p_corr + geom_tile() +
    facet_grid(~ type, labeller = label_parsed) +
    scale_x_discrete(expand = c(0,0)) +
    scale_y_discrete(expand = c(0,0)) +
    scale_fill_gradient2(low="#F2AD00", high="#5BBCD6" , mid="white",
                    limits = c(-1,1),
                    breaks = c(-1, -0.5, 0, 0.5, 1),
                    labels = c(-1, -0.5, 0, 0.5, 1),
                    guide=guide_colorbar(direction = "horizontal",
                                         title.position="top",
                                         title.hjust =0.5)) +
    xlab(" ") +
    ylab(" ") +
    theme_bw() +
    coord_fixed() +
    theme(axis.text.x = element_text(angle=90, vjust=0.5),
            legend.position = "bottom",
            strip.background = element_rect(fill="white", color="white"),
            axis.text  =element_text(size=8),
            legend.title  =element_text(size=8),
            legend.text  =element_text(size=8),
            strip.text  =element_text(size=8))
print(p_corr)
```

The phenotypes, kinship, non-genetic covariates and the intermediate phenotype components (such as shared and independendent genetic variant effects) are saved to datadir. In addition to saving all components in .csv format, we also specify gemma as an output format. This option will create phenotype, kinship and covariate files that can directly be used as input for GEMMA.

```
# Save phenotypes, kinship and non-genetic covariates in csv and
# GEMMA-specific format
outdirectory <- savePheno(simulation, directory = datadir,
                            intercept_gemma=TRUE, format=c("csv", "gemma"),
                            saveIntermediate=TRUE)
```

As the simulated genotype dataset was large, we chose to sample from the number of variants on the causal chromosomes and did not read the entire genotype data set into memory. As such, the genotypes themselves

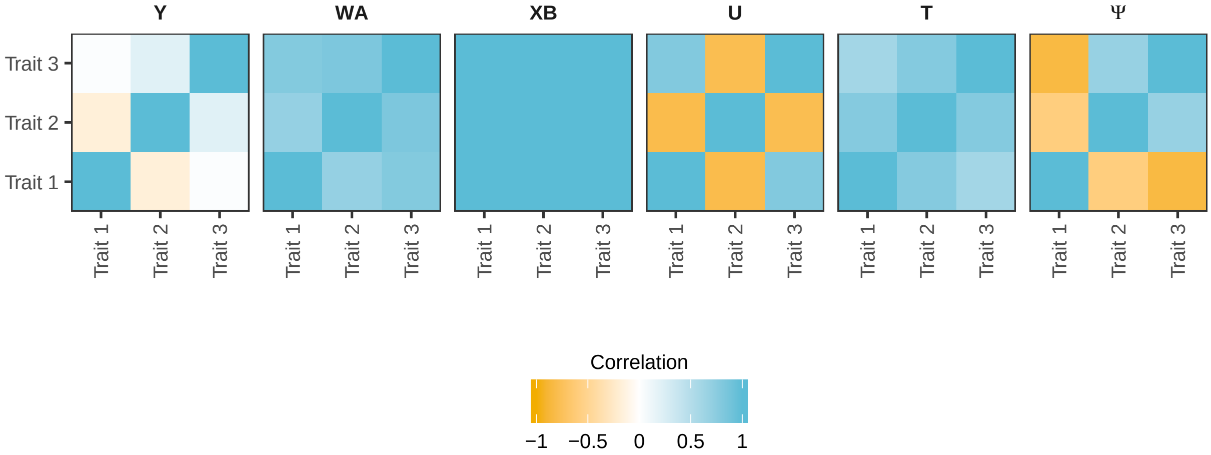

Figure 1: Heatmaps of the trait-by-trait correlation (Pearson correlation) of the simulated phenotype and its five phenotype components.

are not yet in GEMMA specific format. The following code chunk (obtained from the GEMMA user manual) reformats the oxford format (.gen/.sample), to the GEMMA (or BIMBAM format):

```
for chr in `seq 1 22`; do
    cat  $hapdir/genotypes_chr${chr}_hapgen.controls.gen | awk -v s=1000 \
        '{ printf $2 "," $4 "," $5;
        for(i=1; i<=s; i++) printf "," $(i*3+3)*2+$(i*3+4); printf "\n"}' \
        > $hapdir/genotypes_chr${chr}_hapgen.controls.gemma

done
```

Genome-wide association study via GEMMA

One application of *PhenotypeSimulator* is generating phenotypes for model evaluation. As a case study, we show the use of the simulated phenotypes to evaluate the power of multivariate (all phenotypes are analysed jointly) and univariate models (each phenotype is analyses separately) for GWAS where multiple traits per individual are available.

The univariate and multivariate linear mixed model as implemented in GEMMA [3] are briefly described below.

Univariate linear mixed model

In the univariate linear mixed model, the $N \times 1$ vector of a single phenotype from N samples is modeled as the sum of a genetic fixed effect with the $N \times 1$ vector of genetic variant $\mathbf{x}$ and its effect size β, $\mathbf{W} = (\mathbf{w}_1, ;\mathbf{w}_c)$ is an $N \times C$ matrix of C covariates (fixed effects) including a column of 1s (implemented in *PhenotypeSimulator* via intercept_gemma=TRUE); α is a C-vector of the corresponding coefficients including the intercept; $\mathbf{u}$ is an N-vector of random genetic effects; $\mathbf{e}$ is an N-vector of observational noise. σ_g and σ_e are the variance of the genetic and nosie random effects; $\mathbf{K}$ is the known $N \times N$ relatedness matrix and $\mathbf{I}_n$ is an $N \times N$ identity matrix. MVN_N denotes the N-dimensional multivariate normal distribution.

$$\mathbf{y} = \mathbf{x}\beta + \mathbf{W}\alpha + \mathbf{u} + \mathbf{e}$$

with

$$\mathbf{u} \sim MVN_N(0, \sigma_g \mathrm{K})$$

and

$$\mathbf{e} \sim MVN_N(0, \sigma_e I_N)$$

Multivariate linear mixed model

The univariate linear mixed model can be extended to the multivariate linear mixed model with:

$$\mathbf{Y} = x\beta^T + \mathbf{WA} + \mathbf{U} + \mathbf{E}$$

with

$$\mathbf{U} \sim MN_{N\times P}(0, \mathbf{K}, \mathbf{V}_g)$$

and

$$\mathbf{E} \sim MN_{N\times P}(0, \mathbf{I}_N, \mathbf{V}_e)$$

where $\mathbf{Y}$ is the $N \times P$ vector of P phenotype from N samples, $\mathbf{x}$ the $N \times 1$ vector of genetic variant and its $P \times 1$ effect size vector β. $\mathbf{W} = (\mathbf{w}_1, ;\mathbf{w}_c)$ is an $N \times C$ matrix of C covariates (fixed effects) including a column of 1s; A is the $C \times P$-matrix of the corresponding coefficients including the intercept; $\mathbf{U}$ is an $N \times P$-matrix of random genetic effects; $\mathbf{E}$ is an $N \times P$-matrix of observational noise. $\mathbf{V}_g$ and $\mathbf{V}_e$ are the $P \times P$ symmetric matrices of the genetic and noise variance components; $\mathbf{K}$ is the known $N \times N$ relatedness matrix and $\mathbf{I}_n$ is an $N \times N$ identity matrix. $MN(\mu, \mathbf{V}_1, \mathbf{V}_2)$ denotes a matrix normal distribution with mean $= \mu$, the column covariance $\mathbf{V}_1$ and the row covariance $\mathbf{V}_2$.

We use these models for the multi-trait analyses of the three simulated phenotypes (multivariate linear mixed model, flags: -lmm 2 and -n 1 2 3) and the independent analyses of each of the three phenotypes (univariate linear mixed model, flags: -lmm 2 and -n 1 or -n 2 or -n 3). We disable the automatic minor allele frequency filtering by setting -maf 0. The following code chunk shows the GEMMA (version 0.96) setup to run these models on a per chromosome basis

```
hapdir=/homes//hannah/data/hmeyer/Supplementary/CEU.0908.impute.files
datadir=/homes/hannah/tmp/phenotypeSimulator

for chr in `seq 1 22`; do
    # multivariate linear mixed model across all three traits
    gemma0.96 \
    -g $hapdir/genotypes_chr${chr}_hapgen.controls.gemma \
    -p $datadir/Ysim_gemma.txt \
    -d $hapdir/genotypes_eigenvalues \
    -u $hapdir/genotypes_eigenvectors \
    -c $datadir/Covs_gemma.txt \
    -maf 0 \
    -km 1 \
    -lmm 2 \
    -n 1 2 3 \
    -outdir $datadir \
    -o GWAS_gemma_LMM_mt_chr$chr

    # univariate linear mixed model for trait 1
    gemma0.96 \
    -g $hapdir/genotypes_chr${chr}_hapgen.controls.gemma \
    -p $datadir/Ysim_gemma.txt \
    -d $hapdir/genotypes_eigenvalues \
    -u $hapdir/genotypes_eigenvectors \
    -c $datadir/Covs_gemma.txt \
    -maf 0 \
    -km 1 \
```

```
    -lmm 2 \
    -n 1 \
    -outdir $datadir \
    -o GWAS_gemma_LMM_st_trait1_chr$chr

    # univariate linear mixed model for trait 2
     gemma0.96 \
    -g $hapdir/genotypes_chr${chr}_hapgen.controls.gemma \
    -p $datadir/Ysim_gemma.txt \
    -d $hapdir/genotypes_eigenvalues \
    -u $hapdir/genotypes_eigenvectors \
    -c $datadir/Covs_gemma.txt \
    -maf 0 \
    -km 1 \
    -lmm 2 \
    -n 2 \
    -outdir $datadir \
    -o GWAS_gemma_LMM_st_trait2_chr$chr

    # univariate linear mixed model for trait 3
    gemma0.96 \
    -g $hapdir/genotypes_chr${chr}_hapgen.controls.gemma \
    -p $datadir/Ysim_gemma.txt \
    -d $hapdir/genotypes_eigenvalues \
    -u $hapdir/genotypes_eigenvectors \
    -c $datadir/Covs_gemma.txt \
    -maf 0 \
    -km 1 \
    -lmm 2 \
    -n 3 \
    -outdir $datadir \
    -o GWAS_gemma_LMM_st_trait3_chr$chr
done
```

Subsequently, we extract the columns of interest for the power analysis (simply based on p-values). The last column contains the p-value of th analyses, the second column contains the genetic variant identifier. We extract these columns and write the per-chromosome files into a genome-wide file.

```
columns_LMM_st=`head -1 $datadir/GWAS_gemma_LMM_st_trait3_chr22.assoc.txt|wc -w`
columns_LMM_mt=`head -1 $datadir/GWAS_gemma_LMM_mt_chr22.assoc.txt |  wc -w`

for chr in `seq 1 22`; do
    tail -n +2 $datadir/GWAS_gemma_LMM_st_trait1_chr${chr}.assoc.txt | cut \
            -f 2,$columns_LMM_st >> $datadir/GWAS_gemma_LMM_st_trait1_pvalues.txt
    tail -n +2 $datadir/GWAS_gemma_LMM_st_trait2_chr${chr}.assoc.txt | cut \
            -f 2,$columns_LMM_st >> $datadir/GWAS_gemma_LMM_st_trait2_pvalues.txt
    tail -n +2 $datadir/GWAS_gemma_LMM_st_trait3_chr${chr}.assoc.txt | cut \
            -f 2,$columns_LMM_st >> $datadir/GWAS_gemma_LMM_st_trait3_pvalues.txt
    tail -n +2 $datadir/GWAS_gemma_LMM_mt_chr${chr}.assoc.txt | cut \
            -f 2,$columns_LMM_mt >> $datadir/GWAS_gemma_LMM_mt_pvalues.txt
done
```

The quantile-quantile plots of p-values observed from the univariate and multivariate LMMs fitted to all genome-wide SNPs are depicted in Figure 2.

```r
datadir="/homes/hannah/tmp/phenotypeSimulator"

causalSNPs <- fread(paste(datadir, "/SNP_NrSNP10.csv", sep=""),
                    header=TRUE, sep=",", stringsAsFactors=FALSE,
                    data.table=FALSE)
causalSNPnames <- colnames(causalSNPs)

LMM_mt <- fread(paste(datadir, "/GWAS_gemma_LMM_mt_pvalues.txt", sep=""),
                data.table=FALSE, header=FALSE, stringsAsFactors=FALSE)
LMM_st_trait1 <- fread(paste(datadir, "/GWAS_gemma_LMM_st_trait1_pvalues.txt",
                            sep=""),
                data.table=FALSE, header=FALSE, stringsAsFactors=FALSE)
LMM_st_trait2 <- fread(paste(datadir, "/GWAS_gemma_LMM_st_trait2_pvalues.txt",
                            sep=""),
                data.table=FALSE, header=FALSE, stringsAsFactors=FALSE)
LMM_st_trait3 <- fread(paste(datadir, "/GWAS_gemma_LMM_st_trait3_pvalues.txt",
                            sep=""),
                data.table=FALSE, header=FALSE, stringsAsFactors=FALSE)

LMM_mt <- LMM_mt[order(LMM_mt[,2]),]
LMM_mt$expected <- stats::ppoints(nrow(LMM_mt))
LMM_mt$model <- "mvLMM"
LMM_mt$trait <- "all"
colnames(LMM_mt)[1:2] <- c("rsID", "observed")

LMM_st_trait1 <- LMM_st_trait1[order(LMM_st_trait1[,2]),]
LMM_st_trait1$expected <- LMM_mt$expected
LMM_st_trait1$model <- "uvLMM"
LMM_st_trait1$trait <- "trait1"
colnames(LMM_st_trait1)[1:2] <- c("rsID", "observed")

LMM_st_trait2 <- LMM_st_trait2[order(LMM_st_trait2[,2]),]
LMM_st_trait2$expected <- LMM_mt$expected
LMM_st_trait2$model <- "uvLMM"
LMM_st_trait2$trait <- "trait2"
colnames(LMM_st_trait2)[1:2] <- c("rsID", "observed")

LMM_st_trait3 <- LMM_st_trait3[order(LMM_st_trait3[,2]),]
LMM_st_trait3$expected <- LMM_mt$expected
LMM_st_trait3$model <- "uvLMM"
LMM_st_trait3$trait <- "trait3"
colnames(LMM_st_trait3)[1:2] <- c("rsID", "observed")

pvalues <- rbind(LMM_mt, LMM_st_trait1, LMM_st_trait2, LMM_st_trait3)
pvalues$causal <- "all"
pvalues$causal[which(pvalues$rsID %in% causalSNPnames)] <- "simulated causal"
pvalues$causal <- factor(pvalues$causal, levels=c("all", "simulated causal"))

p_qq <- ggplot(data=pvalues,
                aes(x=-log10(expected), y=-log10(observed)))
p_qq <- p_qq + geom_point(aes(color=causal, shape=trait)) +
    scale_color_manual(values=c('darkgrey', '#1b9e77'),
                       name="SNPs",
```

```
                    guide=guide_legend(nrow = 2, title.position="top",
                                       title.hjust =0.5,
                                       override.aes = list(shape=21))) +
    scale_shape_manual(values=c(18, 15:17),
                       name="Traits",
                    guide=guide_legend(nrow = 2,
                                       override.aes = list(colour='darkgrey'),
                                       title.position="top",
                                       title.hjust =0.5)) +
    geom_abline(intercept=0, slope=1, col="black") +
    geom_point(data=dplyr::filter(pvalues, causal == "simulated causal"),
               color='#1b9e77', aes(shape=trait)) +
    xlab(expression(Expected~-~log[10](italic(p))) ) +
    ylab(expression(Observed~-~log[10](italic(p)))) +
    facet_grid(~ model) +
    theme_bw() +
    theme(strip.background = element_rect(fill="white"),
            axis.title = element_text(size=12),
            axis.text  =element_text(size=10),
            legend.title  =element_text(size=12),
            legend.text  =element_text(size=10),
            strip.text  =element_text(size=10),
            legend.position = "bottom",
            legend.direction = "horizontal")
```

Counting the number of causal SNPs that both models can recover, we see that the multi-trait GWAS shows greater power compared to any of the single trait analyses. Here a total of four causal SNPs (Figure 2) compared the combined count of the single-trait GWAS of three was detected with a p-values of less than the commonly applied GWAS threshold of 5×10^{-8} (Table 1). The p-values of the three univariate analyses were adjusted for multiple hypothesis testing (Bonferroni adjustment).

```
sigSNPs <- dplyr::filter(pvalues, causal == "simulated causal",
                         observed < 5*10^-8)
sigSNPs$observed_adjusted <- sigSNPs$observed
sigSNPs$observed_adjusted[which(sigSNPs$trait != "all")] <-
    3*sigSNPs$observed[which(sigSNPs$trait != "all")]
sigSNPs <- dplyr::filter(sigSNPs, observed_adjusted < 5*10^-8)
```

Table 1: P-values from multi-trait GWAS (model == mvLMM) and single-trait GWAS (model == uvLMM)

rsID	model	trait	observed_adjusted
rs6948782	mvLMM	all	3.202576e-30
rs28788872	mvLMM	all	8.465234e-24
5-7362997	mvLMM	all	3.824327e-17
rs10953472	mvLMM	all	7.711818e-10
rs6948782	uvLMM	trait2	4.178292e-18
rs28788872	uvLMM	trait2	2.721007e-14
5-7362997	uvLMM	trait2	6.963468e-10

The ability of linear (mixed) models to detect the SNPs for which a phenotype effect was modelled depends on the allele frequencies of these SNPs and the effect size [4],[5]: the higher the effect size and/or the allele frequencies the better the power to detect the SNP effects. The p-values of all SNPs with simulated effect on

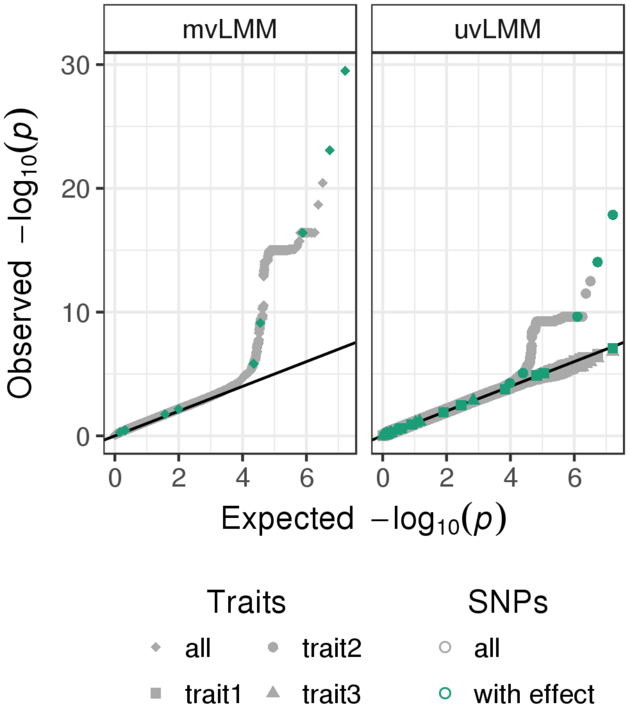

Figure 2: Quantile-quantile plots of p-values observed from the multivariate linear mixed model (mvLMM, traits:all) and the univariate linear mixed models (uvLMM, traits: trait1/trait2/trait3) fitted to each of about eight million genome-wide SNPs (grey), including the ten SNPs for which a phenotype effect was modelled (green).

the phenotypes in relation to their allele frequencies and simulated effect sizes are depicted in Figure 3. There is a strong trend for SNPs with high allele frequencies and large simulated effect sizes to have low p-values.

```
# Read the SNPs simulated to have an effect on the phenotype and their SNP
# effects
SNPs <- read.csv(paste(datadir,"/SNP_NrSNP10.csv", sep=""),
                 row.names=1)
SNPeffect <- read.csv(paste(datadir,"/SNP_effects_NrSNP10.csv", sep=""),
                      row.names=1)


# HapGen simulated SNPs might be encoded as 7-25481146 , which R converts to
# X7.25481146 via make.names in read.table; substitute X and . to get original
# names
SNPnames <- gsub("\\.", "-", gsub("X", "", colnames(SNPs)))
SNPnames <- SNPnames[order(SNPnames)]
simulated_withEffect <- dplyr::filter(pvalues, rsID %in% SNPnames)
```

```
# get the allele frequencies of the SNPs simulated to have an effect on the
# phenotype
freq <- apply(SNPs, 2, getAlleleFrequencies)
minor <- apply(freq, 2, min)
minor <- minor[order(gsub("X", "", names(minor)))]

# format the effect size table and combine with p-value and frequency
# information
effects <- reshape2::melt(SNPeffect, value.name="beta", variable.name="name")
#> No id variables; using all as measure variables
effects$type <- gsub('\\..*', '', effects$name)
effects$rsID <- gsub("\\.", "-", gsub('.*_', '', effects$name))
effects$trait <- rep(paste("trait", 1:3, sep=""), 10)
effects <- effects[order(effects$rsID),]
effects <- dplyr::filter(effects, rsID %in% simulated_withEffect$rsID)

simulated_withEffect <- simulated_withEffect[order(simulated_withEffect[, 1]),]
simulated_withEffect$beta <- NA
simulated_withEffect$beta[simulated_withEffect$trait != "all"] <- effects$beta
simulated_withEffect$freq <- rep(minor[which(SNPnames %in% effects$rsID)], each=4)

# plot association p-values in relation to the absolute value of the simulated
# effect sizes and the SNPs allele frequencies
p <- ggplot(dplyr::filter(simulated_withEffect, trait != "all"))
p + geom_point(aes(x=freq, y=-log10(observed), color=abs(beta), shape=trait)) +
    geom_hline(yintercept=-log10(5*10^(-8)), color="darkgrey") +
    scale_color_gradientn(colours=c('#f0f9e8','#ccebc5','#a8ddb5','#7bccc4',
                                    '#4eb3d3','#2b8cbe','#08589e'),
                          name=expression("| simulated" ~beta~ "|")) +
    scale_shape_discrete(name="Phenotype in GWAS") +
    xlab("Allele frequencies") +
    ylab(expression(-log[10](p-value))) +
    theme_bw()
```

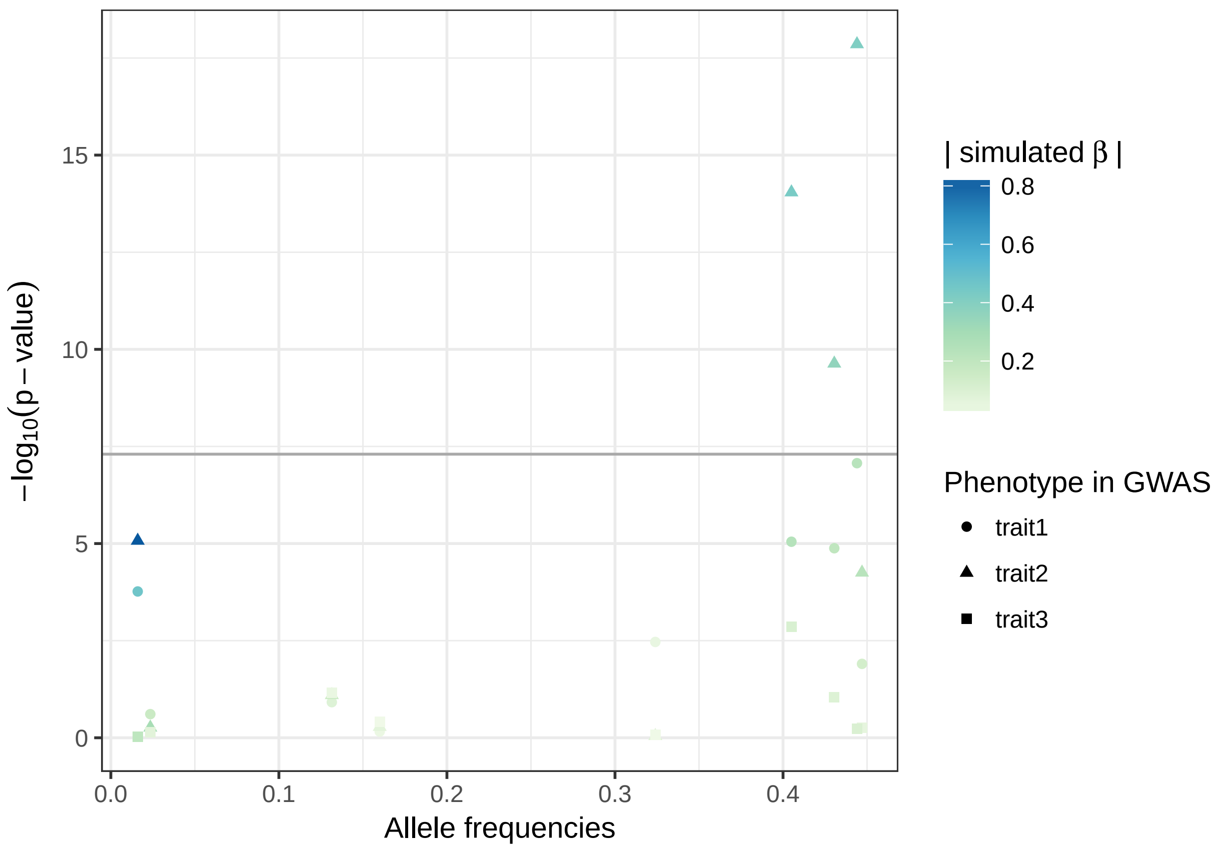

Figure 3: P-values, allele frequencies and simulated effect sizes of the ten SNPs simulated to have an effect on phenotype.

References

1. Su Z, Marchini J, Donnelly P. HAPGEN2: simulation of multiple disease SNPs. Bioinformatics. Oxford University Press; 2011;27: 2304–2305. doi:10.1093/bioinformatics/btr341

2. Chang CC, Chow CC, Tellier LC, Vattikuti S, Purcell SM, Lee JJ. Second-generation PLINK: rising to the challenge of larger and richer datasets. GigaScience. 2015;4: 7. doi:10.1186/s13742-015-0047-8

3. Zhou X, Stephens M. Efficient multivariate linear mixed model algorithms for genome-wide association studies. Nature Methods. 2014;11: 407–9. doi:10.1038/nmeth.2848

4. Cohen J. A power primer. Psychological Bulletin. 1992;112: 155–9. Available: http://www.ncbi.nlm.nih.gov/pubmed/19565683

5. Halsey LG, Curran-Everett D, Vowler SL, Drummond GB. The fickle P value generates irreproducible results. Nature Methods. 2015;12: 179–185. doi:10.1038/nmeth.3288
