# Supplementary material for: PhenotypeSimulator: A comprehensive framework for simulating multi-trait, multi-locus genotype to phenotype relationships
Source: Bioinformatics. 2018 Mar 29;34(17):2951–6. doi: 10.1093/bioinformatics/bty197 (PMC6129313; doi:10.1093/bioinformatics/bty197)
Supplement: Supplementary Data [file bty197_supp.zip › bty197-suppl_data/SimulationBasedOnExampleData.pdf]

Using PhenotypeSimulator to simulate phenotypes based on an example dataset

Hannah Meyer

26/02/2018

PhenotypeSimulator allows for the simulation of high-dimensional phenotypes comprised from a number of different components: genetic variant and population- genetic effects, non-genetic covariates, correlated and observational noise effects. In this vignette, we show the usefulness of being able to specify different phenotype components with distinct properties. We demonstrate how different phenotype components relate directly to a real high-dimensional, image-derived data set of cardiac morphology.

Figure 1 shows the average left ventricular wall thickness of 1,185 healthy volunteers measured at more than 27,000 positions in the left ventricle of the heart [1] [2].

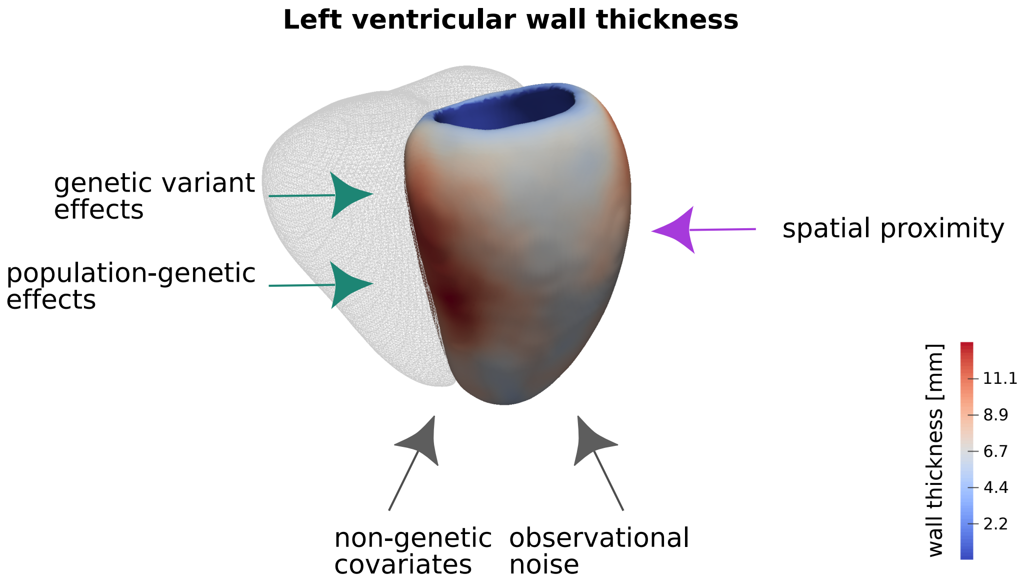

Figure 1: Left ventricular wall thickness and contributing factors. Left ventricular wall thickness was measured at more than 27k positions in a cohort of 1,185 healthy volunteers. The average wall thickness at each position is depicted in saturated colours (the mesh depicts the right ventricle for reference). Heart wall thickness is determined by an interplay of genetic (green arrows) and non-genetic factors (grey arrows). Additional correlation between positional thickness can be observed for measures in close spatial proximity.

These cardiac morphology measurements are likely shaped by a combination of direct genetic variant effects, population genetic effects and non-genetic covariates. In addition, other influences like image acquisition and processing can contribute to the final measurements - these effects are summarised as observational noise. Furthermore, by the nature of the measurements i.e. wall thickness of an approximately smooth ventricular wall, measurements in close spatial proximity will be strongly correlated.

The following sections will show the distribution of additional cohort measurements like height and age (non-genetic covarariates) and the correlation of the measurements by proximity. In analogy to those, similar components will then be simulated with *PhenotypeSimulator*.

Cardiac morphology data

The following section depicts data from 100 randomly selected participants of the 1,185 healthy volunteers of the original study. From the 27k heart wall thickness measurements, a subset of 1,000 measurements ie. phenotypes was chosen to depict the correlation pattern.

```
library(ggplot2)
library(PhenotypeSimulator)

pheno <- readRDS(paste(directory, "/pheno_small.rds", sep=""))
covs <- readRDS(paste(directory, "/covs.rds",sep=""))

dim(pheno)
```

```
## [1] 1185 1000
```

```
dim(covs)
```

```
## [1] 100   5
```

```
colnames(covs)
```

```
## [1] "Sex"    "Age"    "Height" "Weight" "Slices"
```

As excample covariates of the dataset, age, height, weights, sex and the number slices in the 2D cardiac MRI were chosen (see original publication for details on phenotypes [1]). Figure 2 shows the distribution of these covariates across the 100 individuals.

```
covs_melt <- reshape2::melt(covs, value.name="meassure",
                            variable.name="covariate")
```

```
## No id variables; using all as measure variables
```

```
covs_melt$covariate <- factor(covs_melt$covariate,
                              levels=c("Age", "Height", "Weight", "Sex",
                                       "Slices"),
                              labels= c("Age [years]", "Height [cm]",
                                        "Weight [kg]", "Sex", "Slices"))
p_cont <- ggplot(dplyr::filter(covs_melt, covariate %in% c("Age [years]",
                                                           "Height [cm]",
                                                           "Weight [kg]")),
                 aes(x=meassure))
p_cont <- p_cont + geom_histogram(binwidth=1) +
    facet_wrap(~covariate, scales = "free") +
    theme_bw() +
    theme(axis.title.x=element_blank(),
          strip.background = element_rect(fill="white", color="black"))

p_cat <- ggplot(dplyr::filter(covs_melt, covariate %in% c("Sex", "Slices")),
                 aes(x=meassure))
p_cat <- p_cat + geom_bar(aes(x=meassure)) +
    facet_wrap(~covariate, scales = "free") +
    theme_bw() +
    theme(axis.title.x=element_blank(),
          strip.background = element_rect(fill="white", color="black"))

combine <- cowplot::plot_grid(p_cont, p_cat, nrow=2)
print(combine)
```

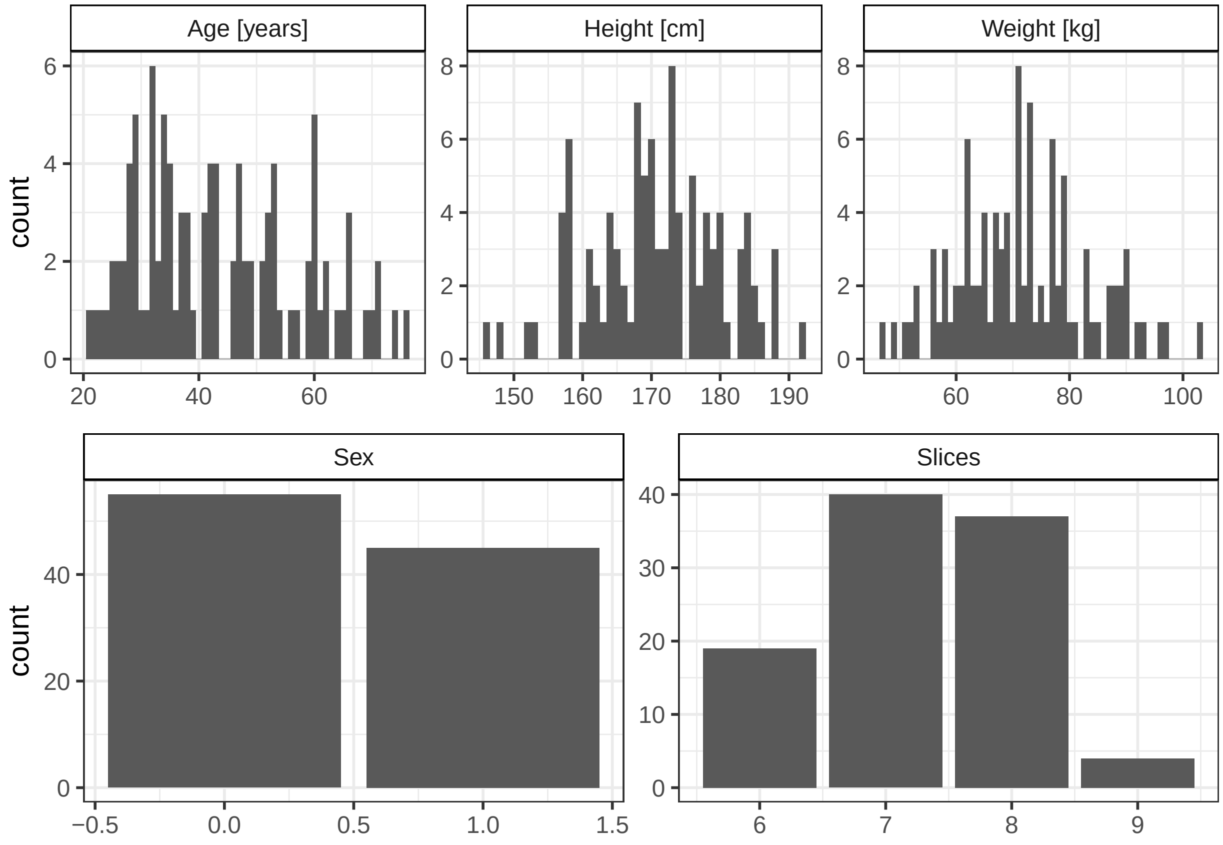

Figure 2: Distribution of covariates. Histograms of the reduced cohort data (100 individuals) for sex, age, height, weight and number of 2D cardiac MRI slices.

The correlation of the 1,000 left-ventricular wall thickness measurements is shown in Figure 3. While we don't know the exact influences of each covariate, the genetic effects or the observational noise, we can clearly see that there is spatial correlation effect, where positions in proximity (close to the diagonal) in general are more correlated than more distant positions.

```
cor_pheno <- cor(pheno)
gplots::heatmap.2(cor_pheno, trace="none", dendrogram="none", keysize=1,
                  col=colorRampPalette(c("white", "#5BBCD6")),
                  labRow=FALSE, labCol=FALSE,
                  breaks=seq(0,1,0.001),
                  key.title="",
                  key.xlab="Pearson's correlation coefficient",
                  key.ylab="",
                  density.info="none")
```

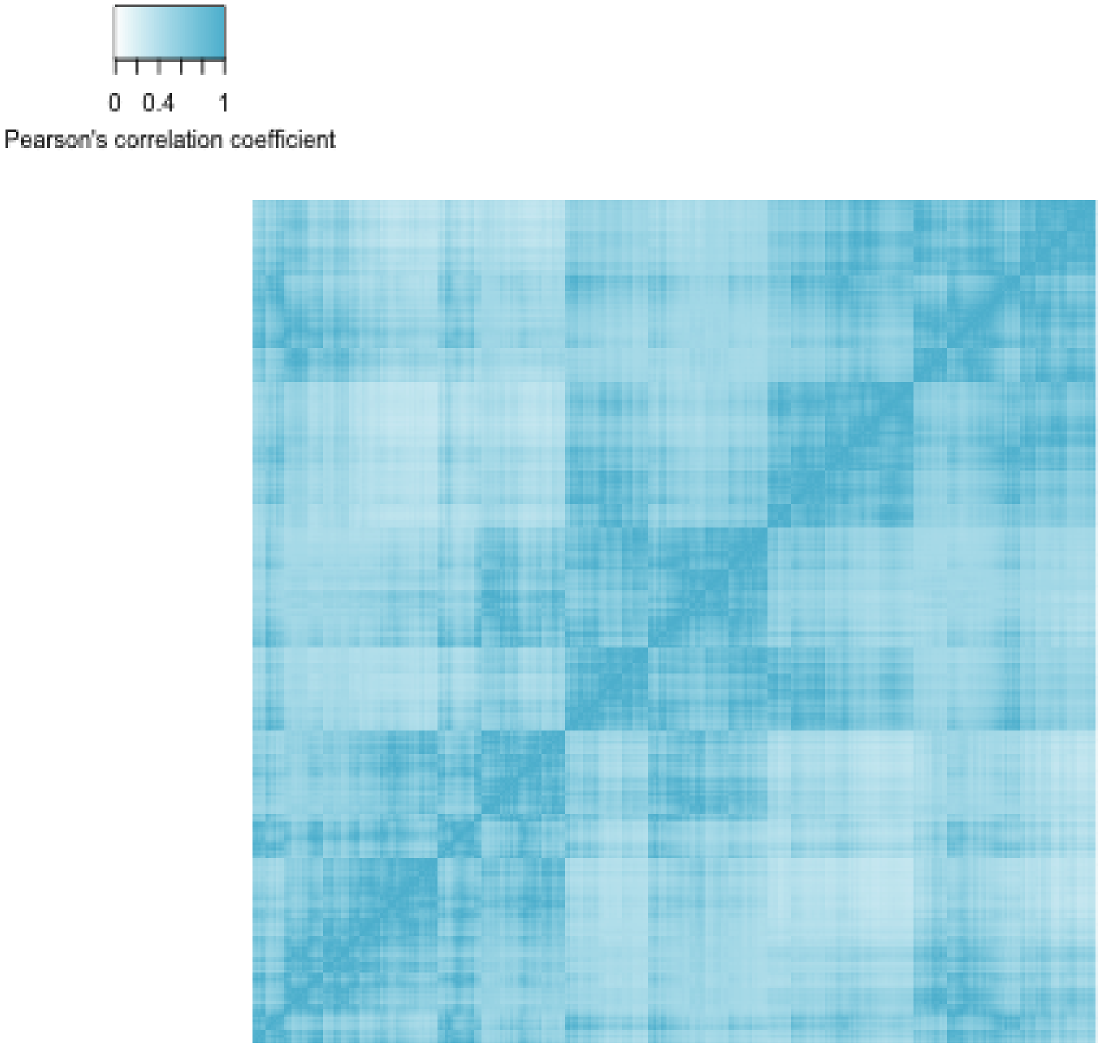

Figure 3: Correlation of left ventricular wall thickness measurements.

Simulation

For the simulation of similar phenotype components as shown above, we make the assumption that the above distributions approximate a uniform (age), normal (weight, height) and binomial (sex) distribution. The slices are assumed to be uniformly drawn from four categories. In the following, we extract the relevant distribution parameters: mean and standard deviation for normal distribution, range for uniform distribution, and proportion of ones from binomial distribution. To approximate the spatial correlation, we get the 0.999 quantile of the correlation values.

```
age_range <- range(covs$Age)
```

```r
weight_mean <- mean(covs$Weight)
weight_sd <- sd(covs$Weight)

height_mean <- mean(covs$Height)
height_sd <- sd(covs$Height)

slices_categories <- length(unique(covs$Slices))

sex_proportion_male <- length(which(covs$Sex==1))/nrow(covs)

correlation <- quantile(unlist(cor_pheno[lower.tri(cor_pheno)]), 0.999)
```

We use these distribution parameters to simulate non-genetic covariate effects with *PhenotypeSimulator::noiseFixedEffects*. For the simulation of the spatial correlation effect, we use the above quantile cut-off for the construction of the correlation matrix with *PhenotypeSimulator::correlatedBgEffects*.

```r
set.seed(25)
covs_simulated <- noiseFixedEffects(N=100, P=1000, NrFixedEffects = 5,
                                    NrConfounders = 1,
                  distConfounders = c("unif", "norm", "norm", "cat_unif", "bin"),
                  mConfounders = c(mean(age_range), weight_mean, height_mean),
                  sdConfounders = c(age_range[2] -mean(age_range), weight_sd,
                                    height_sd),
                  probConfounders = sex_proportion_male,
                  catConfounders = slices_categories)

background_simulated <- correlatedBgEffects(N=100, P=1000, pcorr=correlation)
```

Figure 4 and 5 show the simulated covariates and spatial correlation based on the parameters from the real data. While the distributions are not a perfect replication (as expected with small sample sizes and our initial assumptions about the true underlying distributions of the covariates), they will serve as good approximations for simulating similar datasets as the one we based the simulations on.

```r
covs_simulated_melt <- reshape2::melt(covs_simulated$cov, value.name="meassure",
                           variable.name="covariate")
```

```
## No id variables; using all as measure variables
```

```r
covs_simulated_melt$covariate <- factor(covs_simulated_melt$covariate,
                              levels=c("sharedConfounder1_unif1",
                                       "sharedConfounder2_norm1",
                                       "sharedConfounder3_norm1",
                                       "sharedConfounder5_bin1",
                                       "sharedConfounder4_cat_unif1"),
                              labels= c("Age simulated", "Height simulated",
                                        "Weight simulated", "Sex simulated",
                                        "Slices simulated"))
p_cont <- ggplot(dplyr::filter(covs_simulated_melt, covariate %in%
                                   c("Age simulated", "Height simulated",
                                                        "Weight simulated")),
                   aes(x=meassure))
p_cont <- p_cont + geom_histogram(binwidth=1) +
    facet_wrap(~covariate, scales = "free") +
    theme_bw() +
    theme(axis.title.x=element_blank(),
          strip.background = element_rect(fill="white", color="black"))
```

```r
p_cat <- ggplot(dplyr::filter(covs_simulated_melt, covariate %in%
                                   c("Sex simulated", "Slices simulated")),
                   aes(x=meassure))
p_cat <- p_cat + geom_bar(aes(x=meassure)) +
    facet_wrap(~covariate, scales = "free") +
    theme_bw() +
    theme(axis.title.x=element_blank(),
          strip.background = element_rect(fill="white", color="black"))

combine <- cowplot::plot_grid(p_cont, p_cat, nrow=2)
print(combine)
```

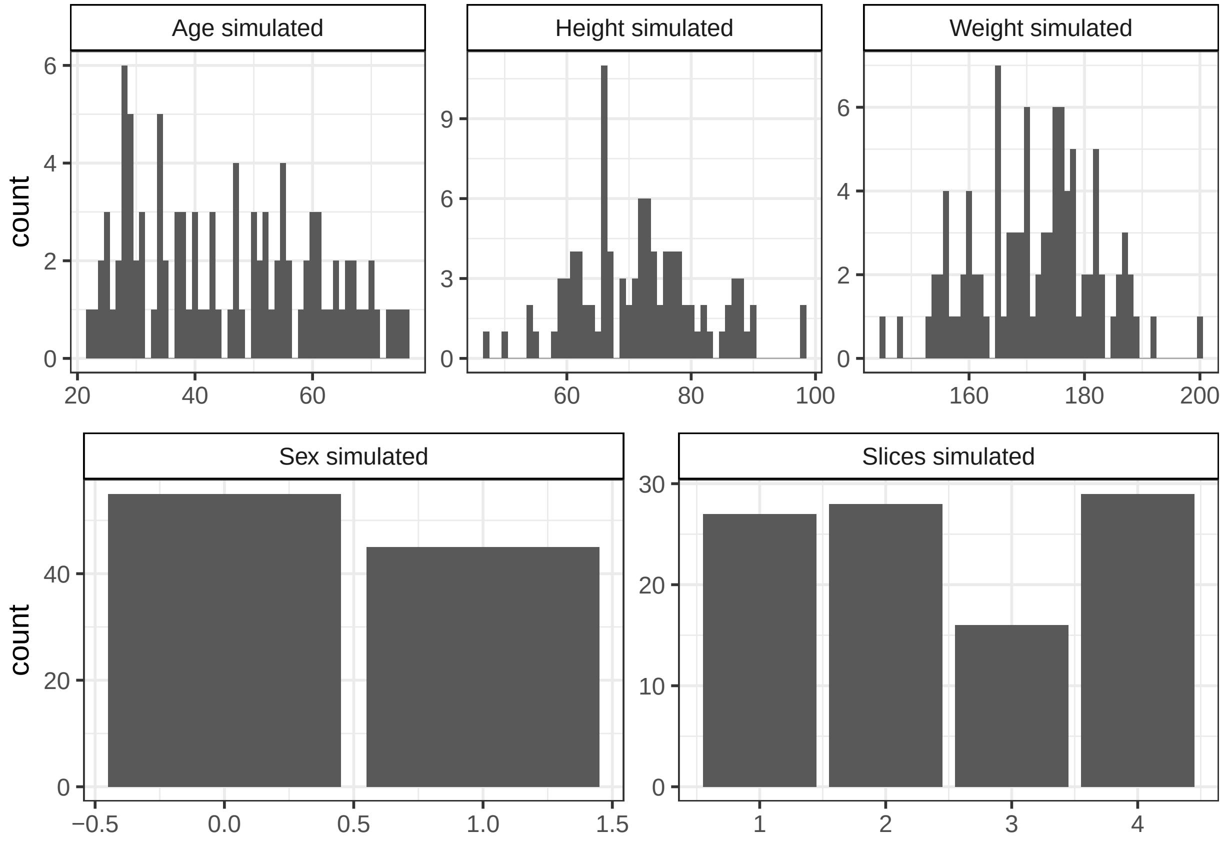

Figure 4: Distribution of simulated covariates.

```r
gplots::heatmap.2(cor(background_simulated$correlatedBg), trace="none",
                  dendrogram="none",
                  keysize=1, col=colorRampPalette(c("white", "#5BBCD6")),
                  labRow=FALSE, labCol=FALSE,
                  breaks=seq(0,1,0.001),
                  key.title="",
                  key.xlab="Pearson's correlation coefficient",
                  key.ylab="",
                  density.info="none")
```

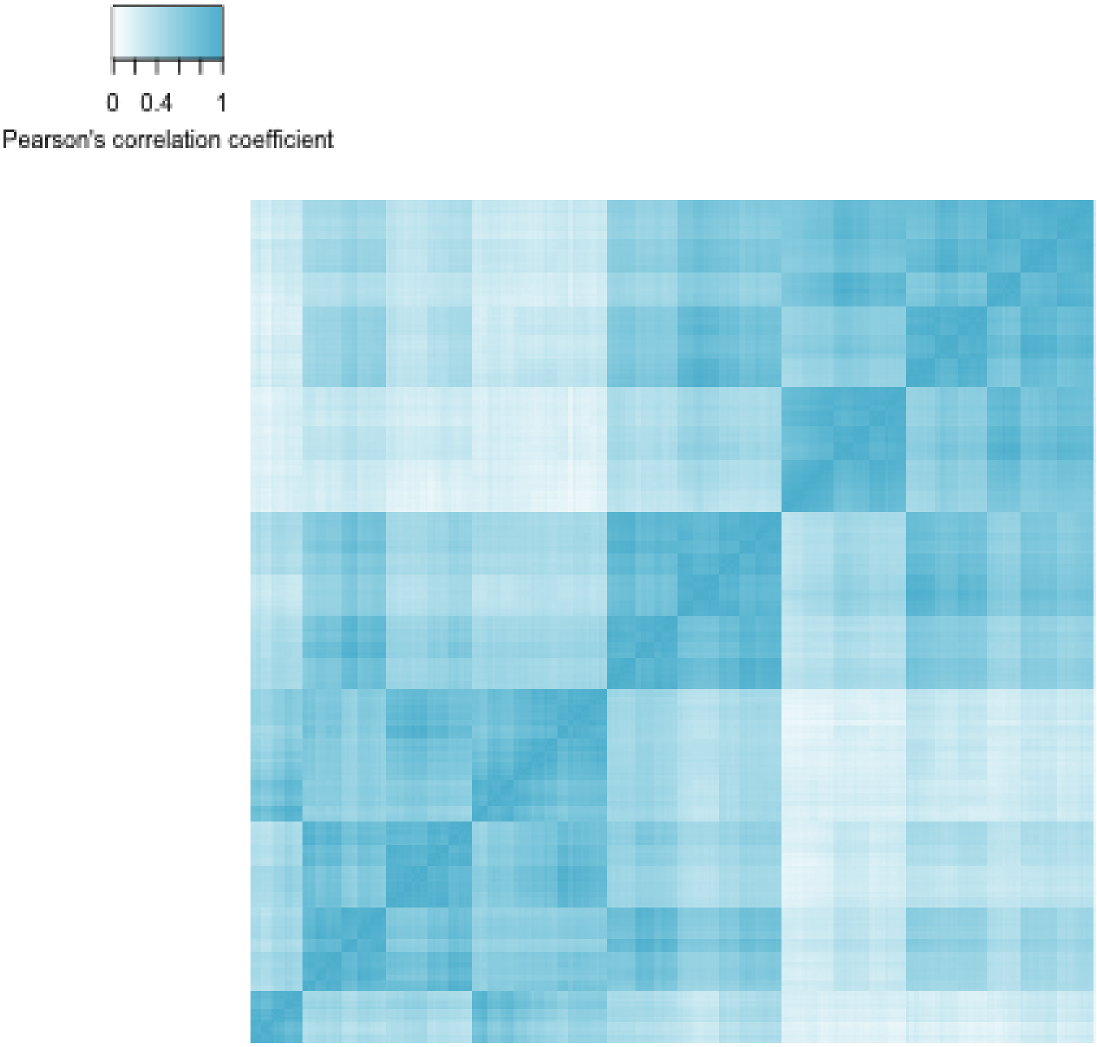

Figure 5: Correlation of simulated spatial proximity.

References

1. Marvao A de, Dawes TJ, Shi W, Minas C, Keenan NG, Diamond T, et al. Population-based studies of myocardial hypertrophy: high resolution cardiovascular magnetic resonance atlases improve statistical power. Journal of Cardiovascular Magnetic Resonance. 2014;16: 16. doi:10.1186/1532-429X-16-16

2. Biffi C, Marvao A de, Attard MI, Dawes TJ, Whiffin N, Bai W, et al. Three-dimensional Cardiovascular Imaging-Genetics: A Mass Univariate Framework. Bioinformatics. 2017; doi:10.1093/bioinformatics/btx552
