# Supplementary material for: PhenotypeSimulator: A comprehensive framework for simulating multi-trait, multi-locus genotype to phenotype relationships
Source: Bioinformatics. 2018 Mar 29;34(17):2951–6. doi: 10.1093/bioinformatics/bty197 (PMC6129313; doi:10.1093/bioinformatics/bty197)
Supplement: Supplementary Data [file bty197_supp.zip › bty197-suppl_data/SimulationSchemeInfinitesimalGeneticEffect.pdf]

1 Simulation scheme for the infinitesimal genetic effects

The following section describes the derivation of the simulation scheme for the infinitesimal genetic effects. A suitable model for simulating the infinitesimal genetic effect $\mathbf{U} \in \mathcal{R}^{N,\ P}$ with known $N \times N$ sample (row) covariance is a matrix-normally distributed random variable, defined by its mean $\mathbf{M} \in \mathcal{R}^{N,\ P}$, its row covariance $\mathbf{D} \in \mathcal{R}^{N,\ N}$ and its column covariance $\mathbf{C} \in \mathcal{R}^{P,\ P}$:

$$\mathbf{U} \sim \mathcal{MN}_{N,P}\left(\mathbf{M},\ \mathbf{D},\ \mathbf{C}\right). \tag{1}$$

With the $N \times N$ sample-by-sample covariance captured in the genetic kinship matrix K and $\mathbf{M} = 0$, the component of $\mathbf{U}$ which has to be simulated is the trait-by-trait covariance $\mathbf{C}$:

$$\mathbf{U} \sim \mathcal{MN}_{N,P}\left(\mathbf{0},\ \mathbf{K},\ \mathbf{C}\right) \tag{2}$$

The structure of $\mathbf{C}$ depends on the design of the covariance effect. In order to simulate $\mathbf{C}$, $\mathbf{G}$ is first expressed in terms of a multivariate normal distribution

$$\text{vec}(\mathbf{U}) \sim \mathcal{N}_{N\times P}\left(\mathbf{0},\ \mathbf{C}\otimes\mathbf{K}\right). \tag{3}$$

With the Cholesky decomposition of $\mathbf{K}$ and $\mathbf{C}$ into $\mathbf{K} = \mathbf{B}\mathbf{B}^T$ and $\mathbf{C} = \mathbf{A}\mathbf{A}^T$

$$\text{vec}(\mathbf{U}) \sim \mathcal{N}_{N\times P}\left(\mathbf{0},\ \mathbf{A}\mathbf{A}^T\otimes\mathbf{B}\mathbf{B}^T\right), \tag{4}$$

which can be rearranged as

$$\text{vec}(\mathbf{U}) \sim \mathcal{N}_{N\times P}\left(\mathbf{0},\ (\mathbf{A}\otimes\mathbf{B})\mathbf{I}(\mathbf{A}\otimes\mathbf{B})^T)\right). \tag{5}$$

$\mathbf{I}$ is the identity matrix. Using the property of a normally distributed random variable $\mathbf{Y}$ with mean $\boldsymbol{\mu}$ and covariance matrix $\boldsymbol{\Sigma}$

$$w\mathbf{Y} \sim \mathcal{N}\left(w\boldsymbol{\mu},\ w\boldsymbol{\Sigma}w^T\right), \tag{6}$$

we can let $\text{vec}(\mathbf{U}) = (\mathbf{A}\otimes\mathbf{B})\text{vec}(\mathbf{Y})$ and $\mathbf{Y} \sim \mathcal{N}_{N\times P}\left(\mathbf{0},\ \mathbf{I}\right)$ such that

$$(\mathbf{A}\otimes\mathbf{B})\text{vec}(\mathbf{Y}) \sim \mathcal{N}_{N\times P}\left(\mathbf{0},\ (\mathbf{A}\otimes\mathbf{B})\mathbf{I}(\mathbf{A}\otimes\mathbf{B})^T\right) \tag{7}$$

Using (Horn and Johnson, 1985): Lemma 4.3.1, we get

$$(\mathbf{A}\otimes\mathbf{B})\text{vec}(\mathbf{Y}) = \text{vec}(\mathbf{B}\mathbf{Y}\mathbf{A}^T) = \text{vec}(\mathbf{U}). \tag{8}$$

References

Horn, R. A. and Johnson, C. R. (1985). *Matrix analysis*. Cambridge University Press, New York, 23 edition.
