# Supplementary material for: PhenotypeSimulator: A comprehensive framework for simulating multi-trait, multi-locus genotype to phenotype relationships
Source: Bioinformatics. 2018 Mar 29;34(17):2951–6. doi: 10.1093/bioinformatics/bty197 (PMC6129313; doi:10.1093/bioinformatics/bty197)
Supplement: Supplementary Data [file bty197_supp.zip › bty197-suppl_data/SupplementaryTable.pdf]

Table 1: **Software tools for genotype and phenotypes simulation.** The overview of the software tools is continued in the following two tables.

**Tool**	**Reference**	**Genotype simulation**	**Phenotype simulation**
MetaSim	(Strand, 2002)	Demographic and evolutionary models	no
MS	(Hudson, 2002)	WrightFisher neutral model	no
simuPop	(Peng and Kimmel, 2005)	Forward-time simulation	case-control/quantitative traits
CoaSim	(Mailund *et al.*, 2005)	Backward-time simulation	case-control
GENOME	(Liang *et al.*, 2007)	Backward-time simulation	no
HAP-SAMPLE	(Wright *et al.*, 2007)	Re-sampling approach	case-control and affected-child trio
QuantiNemo	(Neuenschwander *et al.*, 2008)	Forward-time simulation	multiple, uncorrelated quantitative traits
ForSim	(Lambert *et al.*, 2008)	Forward-time simulation	uni‘/multivariate quantitative traits
Fregene/SAMPLE	(Chadeau-Hyam *et al.*, 2008)	Forward-time simulation	case-control/one quantitative trait
GenomePop2	(Carvajal-Rodríguez, 2008)	Forward-time simulation	no
simuGWAS	(Peng and Amos, 2010)	Forward-time simulation (based on real data); based on simuPop	case-control/quantitative traits
MSMS	(Ewing and Hermisson, 2010)	Backward-time simulation	no
phenosim	(Günther *et al.*, 2011)	no	case-control/quantitative traits
SimLD	(Yuan *et al.*, 2011)	Population simulation based on LD-level matching	case-control
Hapgen2	(Su *et al.*, 2011)	Re-sampling approach	case-control
MultiPhen	(O'Reilly *et al.*, 2012)	simple binomial distribution with 2 trials	multiple, correlated quantitative traits
SimRare	(Li *et al.*, 2012)	Forward-time simulation	case-control/one quantitative trait
hypred	(Technow, 2014)	Forward-time simulation	quantitative traits (?)
GPOPSIM	(Zhang *et al.*, 2015)	Mutation-Drift Equilibrium Model	case-control/quantitative traits
PLINK	(Chang *et al.*, 2015)	simple binomial distribution with 2 trials	case-control
SeqSIMLA2	(Chung *et al.*, 2015)	Forward-time simulation	case-control/two quantitative traits
msprime	(Kelleher *et al.*, 2016)	Backward-time simulation	no
multiTraitGWASsimulation	(Porter and O'Reilly, 2017)	simple binomial distribution with 2 trials	multiple, correlated quantitative traits
Cophesim	(Zhbannikov *et al.*, 2017)	no	case-control/one quantitative/survival trait

Table 2: **Software tools for genotype and phenotypes simulation-** continued.

**Tool**	**Code**	**Link**
MetaSim	C++	`https://cran.r-project.org/web/packages/rmetasim/vignettes/CreatingLandscapes.html`
MS	C	`http://home.uchicago.edu/rhudson1/source/mksamples.html`
simuPop	Python	`http://simupop.sourceforge.net/Main/HomePage`
CoaSim	C++/Guile Scheme/Python	`http://users-birc.au.dk/mailund/CoaSim/`
GENOME	C++	`http://csg.sph.umich.edu/liang/genome/download.html`
HAP-SAMPLE	C	`https://sites.google.com/a/umich.edu/leeshawn/software`
QuantiNemo	C++	`https://www2.unil.ch/popgen/softwares/quantinemo/`
ForSim	C++	`http://anth.la.psu.edu/research/research-labs/weiss-lab/research/research`
Fregene/SAMPLE	C++	`https://www.ebi.ac.uk/sites/ebi.ac.uk/files/groups/kersey_service_team/FREGEN/documentation_htm`
GenomePop2	C++	`http://acraaj.webs.uvigo.es/GenomePop2.html`
simuGWAS	Python	`http://simupop.sourceforge.net/Cookbook/SimuGWAS`
MSMS	Java	`http://www.mabs.at/ewing/msms/`
phenosim	Python	`http://evoplant.uni-hohenheim.de/downloads/`
SimLD	C++	`https://sourceforge.net/projects/simld/`
Hapgen2	C++	`http://mathgen.stats.ox.ac.uk/genetics_software/hapgen/hapgen2.html`
MultiPhen	R	`https://cran.r-project.org/web/packages/MultiPhen/index.html`
SimRare	Python/C++	`https://code.google.com/archive/p/simrare/`
hypred	R	`https://cran.r-project.org/src/contrib/Archive/hypred/`
GPOPSIM	Fortran	`https://github.com/SCAU-AnimalGenetics/GPOPSIM`
PLINK	C++	`https://www.cog-genomics.org/plink/1.9/`
SeqSIMLA2	C++	`http://seqsimla.sourceforge.net/`
msprime	Python/C	`https://pypi.python.org/pypi/msprime`
multiTraitGWASsimulation	R	`http://multitraitgwas.kcl.ac.uk:3838/sim-app/`
Cophesim	Python	`https://bitbucket.org/izhbannikov/cophesim`

Table 3: **Software tools for genotype and phenotypes simulation-** continued.

**Tool**	**Output format**
MetaSim	chosen by user (in R)
MS	MS-specific output format described in publicationn/manual
simuPop	chosen by user (in python)
CoaSim	xml/txt
GENOME	GENOME-specific output files
HAP-SAMPLE	genotypes and haplotypes in .txt format
QuantiNemo	FSTAT format
ForSim	chosen by user (in python/R/ruby)
Fregene/SAMPLE	xml
GenomePop2	Genepop/Nexus
simuGWAS	chosen by user (in python)
MSMS	MS-like output with some MSMS-specific additional lines
phenosim	standard formats for GWAS analysis (plink, blossoc, emmax, tassel)
SimLD	No output description in publication
Hapgen2	oxgen format (compatible with Impute2/SNPTEST/GTOOLS)
MultiPhen	chosen by user (in R)
SimRare	.ped files
hypred	chosen by user (in R)
GPOPSIM	GPOPSIM-specific output files
PLINK	plink binary .bed, .bim, .fam (genotypes, SNP information, sample information)
SeqSIMLA2	plink binary .bed, .bim, .fam (genotypes, SNP information, sample information)
msprime	msprime history file in HDF5 format
multiTraitGWASsimulation	.txt
Cophesim	EMMAX, BLOSSOC, Plink, QTDT, TASSEL and GenABEL formats

References

Carvajal-Rodríguez, A. (2008). GENOMEPOP: A program to simulate genomes in populations. *BMC Bioinformatics*, **9**(1), 223.

Chadeau-Hyam, M., Hoggart, C. J., O'Reilly, P. F., Whittaker, J. C., De Iorio, M., and Balding, D. J. (2008). Fregene: Simulation of realistic sequence-level data in populations and ascertained samples. *BMC Bioinformatics*, **9**(1), 364.

Chang, C. C., Chow, C. C., Tellier, L. C., Vattikuti, S., Purcell, S. M., and Lee, J. J. (2015). Second-generation PLINK: rising to the challenge of larger and richer datasets. *GigaScience*, **4**(1), 7.

Chung, R.-H., Tsai, W.-Y., Hsieh, C.-H., Hung, K.-Y., Hsiung, C. A., and Hauser, E. R. (2015). SeqSIMLA2: Simulating Correlated Quantitative Traits Accounting for Shared Environmental Effects in User-Specified Pedigree Structure. *Genetic Epidemiology*, **39**(1), 20–24.

Ewing, G. and Hermisson, J. (2010). MSMS: a coalescent simulation program including recombination, demographic structure and selection at a single locus. *Bioinformatics*, **26**(16), 2064–5.

Günther, T., Gawenda, I., and Schmid, K. J. (2011). phenosim - A software to simulate phenotypes for testing in genome-wide association studies. *BMC Bioinformatics*, **12**(1), 265.

Hudson, R. R. (2002). Generating samples under a Wright-Fisher neutral model of genetic variation. *Bioinformatics*, **18**(2), 337–338.

Kelleher, J., Etheridge, A. M., and McVean, G. (2016). Efficient Coalescent Simulation and Genealogical Analysis for Large Sample Sizes. *PLOS Computational Biology*, **12**(5), e1004842.

Lambert, B. W., Terwilliger, J. D., and Weiss, K. M. (2008). ForSim: a tool for exploring the genetic architecture of complex traits with controlled truth. *Bioinformatics*, **24**(16), 1821–1822.

Li, B., Wang, G., and Leal, S. M. (2012). SimRare: a program to generate and analyze sequence-based data for association studies of quantitative and qualitative traits. *Bioinformatics*, **28**(20), 2703–2704.

Liang, L., Zollner, S., and Abecasis, G. R. (2007). GENOME: a rapid coalescent-based whole genome simulator. *Bioinformatics*, **23**(12), 1565–1567.

Mailund, T., Schierup, M. H., Pedersen, C. N., Mechlenborg, P. J., Madsen, J. N., and Schauser, L. (2005). CoaSim: A flexible environment for simulating genetic data under coalescent models. *BMC Bioinformatics*, **6**(252).

Neuenschwander, S., Hospital, F., Guillaume, F., and Goudet, J. (2008). quantiNemo: an individual-based program to simulate quantitative traits with explicit genetic architecture in a dynamic metapopulation. *Bioinformatics*, **24**(13), 1552–1553.

O'Reilly, P. F., Hoggart, C. J., Pomyen, Y., Calboli, F. C. F., Elliott, P., Jarvelin, M.-R., and Coin, L. J. M. (2012). MultiPhen: Joint Model of Multiple Phenotypes Can Increase Discovery in GWAS. *PLoS ONE*, **7**(5), e34861.

Peng, B. and Amos, C. I. (2010). Forward-time simulation of realistic samples for genome-wide association studies. *BMC Bioinformatics*, **11**(1), 442.

Peng, B. and Kimmel, M. (2005). simuPOP: a forward-time population genetics simulation environment. *Bioinformatics*, **21**(18), 3686–3687.

Porter, H. F. and O'Reilly, P. F. (2017). Multivariate simulation framework reveals performance of multi-trait GWAS methods. *Scientific Reports*, **7**, 38837.

Strand, A. E. (2002). metasim 1.0: an individual-based environment for simulating population genetics of complex population dynamics. *Molecular Ecology Notes*, **2**(3), 373–376.

Su, Z., Marchini, J., and Donnelly, P. (2011). HAPGEN2: simulation of multiple disease SNPs. *Bioinformatics*, **27**(16), 2304–2305.

Technow, F. (2014). hypred: Simulation of Genomic Data in Applied Genetics. *R Package*.

Wright, F. A., Huang, H., Guan, X., Gamiel, K., Jeffries, C., Barry, W. T., Pardo-Manuel de Villena, F., Sullivan, P. F., Wilhelmsen, K. C., and Zou, F. (2007). Simulating association studies: a data-based resampling method for candidate regions or whole genome scans. *Bioinformatics*, **23**(19), 2581–2588.

Yuan, X., Zhang, J., and Wang, Y. (2011). Simulating linkage disequilibrium structures in a human population for SNP association studies. *Biochemical Genetics*, **49**(5-6), 395–409.

Zhang, Z., Li, X., Ding, X., Li, J., and Zhang, Q. (2015). GPOPSIM: a simulation tool for whole-genome genetic data. *BMC Genetics*, **16**(1), 10.

Zhbannikov, I. Y., Arbeev, K. G., Yashin, A. I., and Mitnitski Canada, A. B. (2017). cophesim: a comprehensive phenotype simulator for testing novel association methods [version 1; referees: 2 approved]. *F1000Research*, **6**(1294).
