# Supplementary material for: PhenotypeSimulator: A comprehensive framework for simulating multi-trait, multi-locus genotype to phenotype relationships
Source: Bioinformatics. 2018 Mar 29;34(17):2951–6. doi: 10.1093/bioinformatics/bty197 (PMC6129313; doi:10.1093/bioinformatics/bty197)

Flexible phenotype simulation with PhenotypeSimulator

Hannah Meyer

2018-03-01

Contents

Introduction **1**

Work-flow **2**

Examples **2**

Example 1: Creating a phenotype composed of population structure and observational noise effects only. . . . 2

Example 2: Creating a phenotype composed of five components . . . 4

Command line use **8**

Phenotype component functions **8**

1. Genetic variant effects: . . . 8
2. Infinitesimal genetic effects: . . . 10
3. Non-genetic covariate effects: . . . 11
4. Correlated effects: . . . 12
5. Observational noise effects: . . . 13

Introduction

PhenotypeSimulator allows for the flexible simulation of phenotypes from genetic and noise components. In quantitative genetics, genotype to phenotype mapping is commonly realised by fitting a linear model to the genotype as the explanatory variable and the phenotype as the response variable. Other explanatory variables such as additional sample measures (e.g. age, height, weight) or batch effects can also be included. For linear mixed models, in addition to the fixed effects of the genotype and the covariates, different random effect components can be included, accounting for population structure in the study cohort or observational noise. The application of linear and linear mixed models in quantitative genetics ranges from genetic studies in model organisms such as yeast and *Arabidopsis thaliana* to human molecular, morphological or imaging-derived traits. Developing new methods to efficiently model increasing sample sizes or to apply multi-variate models to sets of phenotypic measurements often requires simulated datasets with a specific underlying phenotype structure.

PhenotypeSimulator allows for the simulation of such phenotypes under different models, including genetic variant effects, infinitesimal genetic effects (reflecting population structure and kinship), non-genetic covariate effects, correlated noise effects and observational noise effects. Different phenotypic effects can be combined into a final phenotype while controling for the proportion of variance explained by each of the components. For each component, the number of variables, their distribution and the design of their effect across traits can be customised. The **work-flow** outlined below summarizes the strategy for the phenotype simulation. In section **Examples**, phenotype simulation for phenotypes with different levels of complexity are demonstrated in both a step-by-step manner or by using the recommended `runSimulation` function. Finally, section **Phenotype component functions** explains the use and simulation strategy of the individual phenotype component-generating functions.

Work-flow

1. Simulate phenotype components of interest:
    1. `geneticFixedEffects`: genetic variant effects,
    2. `geneticBgEffects`: population structure and kinship,
    3. `noiseFixedEffects`: confounding variables e.g sex, age, height…,
    4. `correlatedBgEffects`: additional correlation effects,
    5. `noiseBgEffects`: observational noise;
2. scale components according to variance explained: each phenotype component is scaled to explain a certain proportion of the entire phenotypic variance via `rescaleVariance`;
3. combine phenotype components: rescaled phenotype components are combined to obtain the final simulated phenotype.

`runSimulation` combines the three steps outlined above and allows for the automatic simulation of a phenotype with N number of samples, P number of traits and up to five phenotype components. Alternatively, all components can be simulated, scaled and addedd independently. `savePheno` accepts the output of `runSimulation` to save phenotype components, the final phenotype and -optionally- simulated genotypes in the specified directories.

The following sections outline two examples for phenotype simulations with population structure and observational noise effects and with a more complex set-up of the phenotypes from five phenotype components.

As demonstrated below, each phenotype component function has a number of parameters that allow for customisation of the simulation. `runSimulation` wraps around all these functions and accepts a multitude of parameters. Simple simulation of phenotypes, however, only requires the input of the desired phenotype size (number of samples and traits) and the proportion of variance each phenotype component should take. The recommended use of PhenotypeSimulator is via `runSimulation` as this ensures that all dependencies are automatically set correctly.

The functions used in these examples are explained in detail in **Phenotype component functions**.

Examples

Example 1: Creating a phenotype composed of population structure and observational noise effects only.

Step by step

```
# Set parameters
genVar <- 0.4
noiseVar <- 1- genVar
shared <- 0.6
independent <- 1 - shared

# simulate simple bi-allelic genotypes and estimate kinship
genotypes <- simulateGenotypes(N = 100, NrSNP = 10000,
                               frequencies = c(0.05, 0.1, 0.3, 0.4),
                               verbose = FALSE)
genotypes_sd <- standardiseGenotypes(genotypes$genotypes)
kinship <- getKinship(N=100, X=genotypes_sd, verbose = FALSE)

# simulate phenotype components
genBg <- geneticBgEffects(N = 100, kinship = kinship, P = 15)
```

```r
noiseBg <- noiseBgEffects(N = 100, P = 15)

# rescale phenotype components
genBg_shared_scaled <- rescaleVariance(genBg$shared, shared * genVar)
genBg_independent_scaled <- rescaleVariance(genBg$independent,
                                            independent * genVar)
noiseBg_shared_scaled <- rescaleVariance(noiseBg$shared, shared * noiseVar)
noiseBg_independent_scaled <- rescaleVariance(noiseBg$independent,
                                              independent *noiseVar)

# Total variance proportion shave to add up yo 1
total <- independent * noiseVar + independent * genVar +
    shared * noiseVar + shared * genVar

total == 1
#> [1] TRUE

# combine components into final phenotype
Y <- scale(genBg_shared_scaled$component + noiseBg_shared_scaled$component +
    genBg_independent_scaled$component + noiseBg_independent_scaled$component)
```

via runSimulation

```r
# simulate phenotype with population structure and observational noise effects
# only

# genetic variance
genVar <- 0.4

# random genetic variance: h2b
phenotype <- runSimulation(N = 100, P = 15,  tNrSNP = 10000,
                           SNPfrequencies = c(0.05, 0.1,0.3,0.4),
                           genVar = 0.4, h2bg = 1, phi = 1,
                           verbose = TRUE)
#> Set seed: 219453
#> The total noise variance (noiseVar) is: 0.6
#> The noise model is: noiseBgOnly
#> Proportion of observational noise variance (phi): 1
#> Variance of shared observational noise effect (alpha): 0.8
#>
#> The total genetic variance (genVar) is: 0.4
#> The genetic model is: geneticBgOnly
#> Proportion of variance of infinitesimal genetic effects (h2bg): 1
#> Proportion of variance of shared infinitesimal genetic effects (eta): 0.8
#>
#> Simulate genetic effects (genetic model: geneticBgOnly)
#> Simulate 10000 SNPs...
#> Standardising the 10000 SNPs provided
#> Estimating kinship from 10000 SNPs provided
#> Normalising kinship
#> Simulate infinitesimal genetic effects
#> Simulate noise terms (noise model: noiseBgOnly)
```

```
#> Simulate observational noise effects
#> Construct final simulated phenotype
```

Example 2: Creating a phenotype composed of five components

The phenoytpes to be simulated are composed of genetic variant effects, infinitesimal genetic effects (reflecting population structure and kinship), non-genetic covariate effects, correlated noise effects and observational noise effects.

step-by- step

```
# read genotypes from external file
# use one of the sample genotype file provided in the
# extdata/genotypes/subfolders (e.g.extdata/genotypes/hapgen )
genotypefile <- system.file("extdata/genotypes/hapgen",
                            "genotypes_hapgen.controls.gen",
                            package = "PhenotypeSimulator")
# remove the .gen ending (oxgen specific endings .gen and .sample are added
# automatically )
genotypefile <- gsub("\\.gen","", genotypefile)

genotypes <- readStandardGenotypes(N=100, filename = genotypefile,
                                   format="oxgen",
                                   delimiter = ",", verbose=TRUE)

genotypes_sd <- standardiseGenotypes(genotypes$genotypes)

# kinship estimate based on standardised SNPs
kinship <- getKinship(N=100, X=genotypes_sd, verbose = FALSE)

# simulate 30 genetic variant effects (from non-standardised SNP genotypes)
causalSNPs <- getCausalSNPs(N=100, genotypes = genotypes$genotypes,
                            NrCausalSNPs = 30, verbose = FALSE)
genFixed <- geneticFixedEffects(N = 100, P = 15, X_causal = causalSNPs)

# simulate infinitesimal genetic effects
genBg <- geneticBgEffects(N=100, kinship = kinship, P = 15)

# simulate 4 different confounder effects:
# * 1 binomial covariate effect shared across all traits
# * 2 categorical (3 categories) independent covariate effects
# * 1 categorical (4 categories) independent  covariate effect
# * 2 normally distributed independent and shared covariate effects
noiseFixed <- noiseFixedEffects(N = 100, P = 15, NrFixedEffects = 4,
                                NrConfounders = c(1, 2, 1, 2),
                                pIndependentConfounders = c(0, 1, 1, 0.5),
                                distConfounders = c("bin", "cat_norm",
                                                    "cat_unif", "norm"),
                                probConfounders = 0.2,
                                catConfounders = c(3, 4))
```

```r
# simulate correlated effects with max correlation of 0.8
correlatedBg <- correlatedBgEffects(N = 100, P = 15, pcorr = 0.8)

# simulate observational noise effects
noiseBg <- noiseBgEffects(N = 100, P = 15)

# total SNP effect on phenotype: 0.01
genVar <- 0.6
noiseVar <- 1 - genVar
totalSNPeffect <- 0.01
h2s <- totalSNPeffect/genVar
phi <- 0.6
rho <- 0.1
delta <- 0.3
shared <- 0.8
independent <- 1 - shared

# rescale phenotype components
genFixed_shared_scaled <- rescaleVariance(genFixed$shared, shared * h2s *genVar)
genFixed_independent_scaled <- rescaleVariance(genFixed$independent,
                                                independent * h2s *genVar)
genBg_shared_scaled <- rescaleVariance(genBg$shared, shared * (1-h2s) *genVar)
genBg_independent_scaled <- rescaleVariance(genBg$independent,
                                            independent * (1-h2s) * genVar)

noiseBg_shared_scaled <- rescaleVariance(noiseBg$shared, shared * phi* noiseVar)
noiseBg_independent_scaled <- rescaleVariance(noiseBg$independent,
                                              independent * phi* noiseVar)
correlatedBg_scaled <- rescaleVariance(correlatedBg$correlatedBg,
                                       shared * rho * noiseVar)

noiseFixed_shared_scaled <- rescaleVariance(noiseFixed$shared, shared * delta *
                                              noiseVar)
noiseFixed_independent_scaled <- rescaleVariance(noiseFixed$independent,
                                                 independent * delta * noiseVar)

# Total variance proportions have to add up yo 1
total <- shared * h2s *genVar +  independent * h2s * genVar +
    shared * (1-h2s) * genVar +   independent * (1-h2s) * genVar +
    shared * phi* noiseVar +  independent * phi* noiseVar +
    rho * noiseVar +
    shared * delta * noiseVar +  independent * delta * noiseVar

total == 1
#> [1] TRUE

# combine components into final phenotype
Y <- scale(genBg_shared_scaled$component + noiseBg_shared_scaled$component +
        genBg_independent_scaled$component + noiseBg_independent_scaled$component +
        genFixed_shared_scaled$component + noiseFixed_shared_scaled$component +
        genFixed_independent_scaled$component + noiseFixed_independent_scaled$component +
        correlatedBg_scaled$component)
```

via runSimulation

```
# simulate phenotype with the same five phenotype components
# and settings as above; display progress via verbose=TRUE
phenotype <- runSimulation(N = 100, P = 15, genotypefile = genotypefile,
    format = "oxgen", cNrSNP = 30, genVar = genVar, h2s = h2s,
    phi = 0.6, delta = 0.3, distBetaGenetic = "unif", mBetaGenetic = 0.5,
    sdBetaGenetic = 1, NrFixedEffects = 4, NrConfounders = c(1,
        2, 1, 2), pIndependentConfounders = c(0, 1, 1, 0.5),
    distConfounders = c("bin", "cat_norm", "cat_unif", "norm"),
    probConfounders = 0.2, catConfounders = c(3, 4), pcorr = 0.8,
    verbose = TRUE)
#> Set seed: 219453
#> The total noise variance (noiseVar) is: 0.4
#> The noise model is: noiseFixedAndBgAndCorrelated
#> Proportion of non-genetic covariate variance (delta): 0.3
#> Proportion of variance of shared non-genetic covariate effects (gamma): 0.8
#> Proportion of non-genetic covariates to have a trait-independent effect (pIndependentConfounders ): [illegible]
#> Proportion of traits influenced by independent non-genetic covariate effects (pTraitIndependentConfo[illegible]
#> Proportion of variance of correlated noise effects (rho): 0.1
#> Proportion of observational noise variance (phi): 0.6
#> Variance of shared observational noise effect (alpha): 0.8
#>
#> The total genetic variance (genVar) is: 0.6
#> The genetic model is: geneticFixedAndBg
#> Proportion of variance of genetic variant effects (h2s): 0.0166666666666667
#> Proportion of variance of shared genetic variant effects (theta): 0.8
#> Proportion of genetic variant effects to have a trait-independent fixed effect (pIndependentGenetic)[illegible]
#> Proportion of traits influenced by independent genetic variant effects (pTraitIndependentGenetic): 0[illegible]
#> Proportion of variance of infinitesimal genetic effects (h2bg): 0.983333333333333
#> Proportion of variance of shared infinitesimal genetic effects (eta): 0.8
#>
#> Simulate genetic effects (genetic model: geneticFixedAndBg)
#> Simulate genetic variant effects
#> Out of 15 total phenotypes, 15 traits will be affected by genetic variant effects
#> Out of these affected traits (15), 3 trait(s) will have independent genetic variant effects
#> Standardising the 1000 SNPs provided
#> Estimating kinship from 1000 SNPs provided
#> Normalising kinship
#> Simulate infinitesimal genetic effects
#> Simulate noise terms (noise model: noiseFixedAndBgAndCorrelated)
#> Simulate correlated background effects
#> Simulate observational noise effects
#> Simulate confounder effects
#> Out of 15 total phenotypes, 15 trait(s) will be affected by the  1  covariate effect
#> Out of 15 total phenotypes, 15 trait(s) will be affected by the  2  covariate effect
#> Out of these affected traits (15), 3 trait(s) will have independent covariate effects
#> Out of 15 total phenotypes, 15 trait(s) will be affected by the  3  covariate effect
#> Out of these affected traits (15), 3 trait(s) will have independent covariate effects
#> Out of 15 total phenotypes, 15 trait(s) will be affected by the  4  covariate effect
#> Out of these affected traits (15), 3 trait(s) will have independent covariate effects
#> Construct final simulated phenotype
```

Proportion of variance of the different phenotype components in the final phenotype:

Table 1: Proportion of variance explained by the different phenotype components

	shared effect	independent effect	total effect
varGenFixed	0.008	0.002	0.01
varGenBg	0.472	0.118	0.59
varNoiseFixed	0.096	0.024	0.12
varNoiseBg	0.192	0.048	0.24
varNoiseCorr	0.040	0.000	0.04
sumProportion	0.808	0.192	1.00

The heatmap images below show the values of the phenotype itself (left) and of the correlation between the phenotypic traits (right). The code to produce the images can be seen below (for all subsequent images of the same type the code is not echo-ed).

```
### show 'image' of phenotype and correlation between phenotypic traits
image(t(phenotype$phenoComponentsFinal$Y), main="Phenotype: [samples x traits]",
      axes=FALSE, cex.main=0.8)
mtext(side = 1, text = "Samples", line = 1)
mtext(side = 2, text = "Traits", line = 1)
image(cor(phenotype$phenoComponentsFinal$Y),
      main="Correlation of traits [traits x traits]", axes=FALSE, cex.main=0.8)
mtext(side = 1, text = "Traits", line = 1)
mtext(side = 2, text = "Traits", line = 1)
```

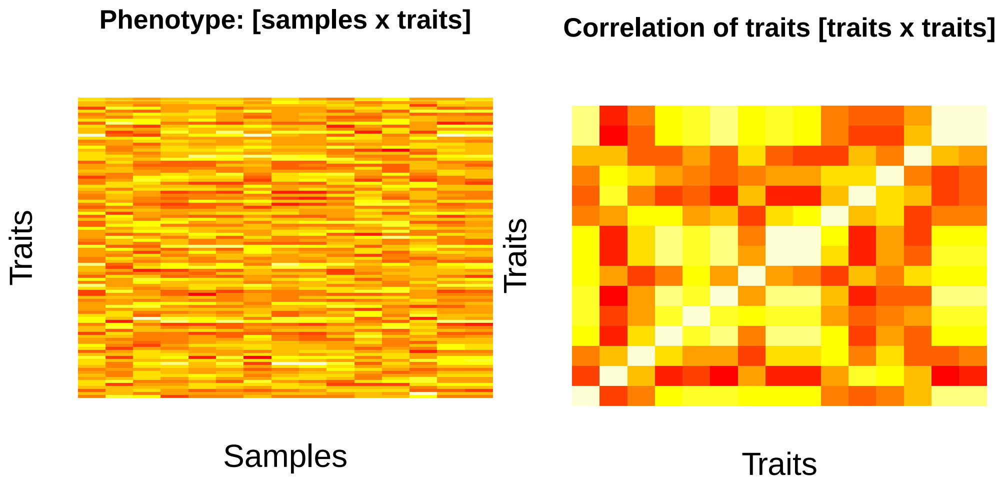

The final phenotype and all its components can be saved via `savePheno`. `savePheno` takes the output of `runSimulation` and saves its components, if saveIntermediate is set to TRUE, intermediate phenotype componentes (such as shared and inpendent variance components are saved); if genotypes were simulated or read from external files and a kinhsip estimated thereof, they will also be saved. The user needs to have writing permission to the specified genotype and phenotype directories. The code below saves the genotypes/phenotypes as .csv files into the subdirectory "test_simulation" of the directories /tmp/genotypes and /tmp/phenotypes. The genotypes are additionally saved in binary plink format, i.e. .bed, .bim and .fam. If the user has writing permissions and the directories do not exist yet, they will be created.

```
out <- savePheno(phenotype, directory="/tmp",
                 outstring="test_simulation",
                 format=c("csv", "plink"), verbose=FALSE)
```

Command line use

PhenotypeSimulator can also be run from the command line via

`Rscript -e "PhenotypeSimulator::simulatePhenotypes()" --args --...` with `--...` being the user supplied simulation paramaters. `Rscript -e "PhenotypeSimulator::simulatePhenotypes()"` takes the same arguments as `runSimulation` and `savePheno`: first, it simulates the specified phenotype components and then saves them into to specified directories. The user will need to have writing permissions to these parent directores. If directoryGeno and directoryPheno do not exist yet, they will be created.

`Rscript -e "PhenotypeSimulator::simulatePhenotypes()" --args --help` will print information about possible input parameters and values they can take on. To generate the same phenotypes as described above via the command line-interface, run the following code from your command line:

```
Rscript -e "PhenotypeSimulator::simulatePhenotypes()" \
--args \
--NrSamples=100 --NrPhenotypes=15 \
--tNrSNP=10000 --cNrSNP=30 \
--SNPfrequencies=0.05,0.1,0.3,0.4 \
--genVar=0.4 --h2s=0.025 --phi=0.6 --delta=0.3 --gamma=1 \
--pcorr=0.8 \
--NrFixedEffects=4 --NrConfounders=1,2,1,2 \
--pIndependentConfounders=0,1,1,0.5 \
--distConfounders=bin,cat_norm,cat_unif,norm \
--probConfounders=0.2 \
--catConfounders=3,4 \
--directory=/tmp \
--subdirectory=test_simulation \
--showProgress \
--saveTable \
--savePLINK
```

Phenotype component functions

1. Genetic variant effects:

Genetic variant effects are simulated as the matrix product of an [N x NrCausalSNPs] genotype matrix and [NrSNP x P] effect size matrix. The genotype matrix can either be drawn from i) a simulated genotype matrix ii) genotypes read from external data or iii) causal SNPs can be randomly sampled as lines from existing genotype files. In the latter case, genotypes are expected to be stored in a [SNPs x N] format, with separate files for each chromosome. The user can either specify which chromosomes to sample the SNPs from or simply provide the total number of chromosomes to sample from. For the simulation of genotypes, the user can specify the `NrSNPs` to simulate and a vector of allele frequencies `frequencies`. These allele frequencies are uniformly sampled and bi-allelic SNPs are simulated, with the sampled allele frequency acting as the probability in a binomial distribution with 2 trials. The example data provided contains the first 500 SNPs (50 samples) on chromosome 22 with a minor allele frequency of less than 2% from the European populations of the the 1000 Genomes project.

```
## a) Draw cuasal SNPs from a simulated genotype matrix
# simulate 10,000 bi-allelic SNP genotypes for 100 samples with randomly drawn
# allele frequencies of 0.05, 0.1, 0.3 and 0.4.
genotypes <- simulateGenotypes(N = 100, NrSNP = 10000,
                               frequencies = c(0.05, 0.1, 0.3,0.4),
                               verbose = FALSE)
```

```r
# draw 10 causal SNPs from the genotype matrix (use non-standardised allele
# codes i.e. (0,1,2))
causalSNPs <- getCausalSNPs(N=100, NrCausalSNPs = 10,
                            genotypes = genotypes$genotypes)
```

```r
## b) Draw SNPs from external genotype files:
# read genotypes from external file
# use one of the sample genotype file provided in the
# extdata/genotypes/subfolders (e.g.extdata/genotypes/hapgen )
genotypefile <- system.file("extdata/genotypes/hapgen",
                            "genotypes_hapgen.controls.gen",
                            package = "PhenotypeSimulator")
# remove the .gen ending (oxbgen specific endings .gen and .sample are added
# automatically )
genotypefile <- gsub("\\.gen","", genotypefile)

genotypes <- readStandardGenotypes(N = 100, filename = genotypefile,
                                   format="oxgen",
                                   delimiter = ",", verbose=TRUE)

causalSNPsFromFile <- getCausalSNPs(N = 100, NrCausalSNPs = 10,
                                   genotypes=genotypes$genotypes)
```

```r
## c) draw 10 causal SNPs from external genotype files: sample 10 SNPs from
## chromosome 22
# use sample genotype file provided in the extdata/genotypes folder
genotypeFile <- system.file("extdata/genotypes/", "genotypes_chr22.csv",
                            package = "PhenotypeSimulator")
genoFilePrefix <- gsub("chr.*", "", genotypeFile)
genoFileSuffix <- ".csv"

causalSNPsSampledFromFile <- getCausalSNPs(N = 10, NrCausalSNPs = 10, chr = 22,
                                   genoFilePrefix = genoFilePrefix,
                                   genoFileSuffix = genoFileSuffix,
                                   delimiter = ",", verbose=TRUE)
#> Draw 10 causal SNPs from 1 chromosomes...
#> Causal chromosomes: 22
#> Get causal SNPs from chromsome-wide SNP files ( /Library/Frameworks/R.framework/Versions/3.4/Resourc
#> Get 10 causal SNPs from chromsome 22 ...
#> Count number of SNPs on chromosome 22 ...
#> Sample SNPs on chromosome 22 ...
#> Sampling 10 samples from 50 genotypes provided
```

The effects attached to the causal SNPs can be classified into two categories: i) a SNP can have a shared effect across traits or an independent effect across traits. The function `geneticFixedEffects` allows the user the specify what proportion `pIndependentGenetic` of SNPs should have independent effects and in the case of an independent effect, the proportion of traits `pTraitIndependentGenetic` affected by the independent effect.

```r
# create genetic variant effects with 20% of SNPs having a specific effect,
# affecting 40% of all simulated traits
fixedGenetic <- geneticFixedEffects(X_causal = causalSNPsFromFile,
                                   N = 100, P = 10,
```

```
                          pIndependentGenetic = 0.2,
                          pTraitIndependentGenetic = 0.4)
```

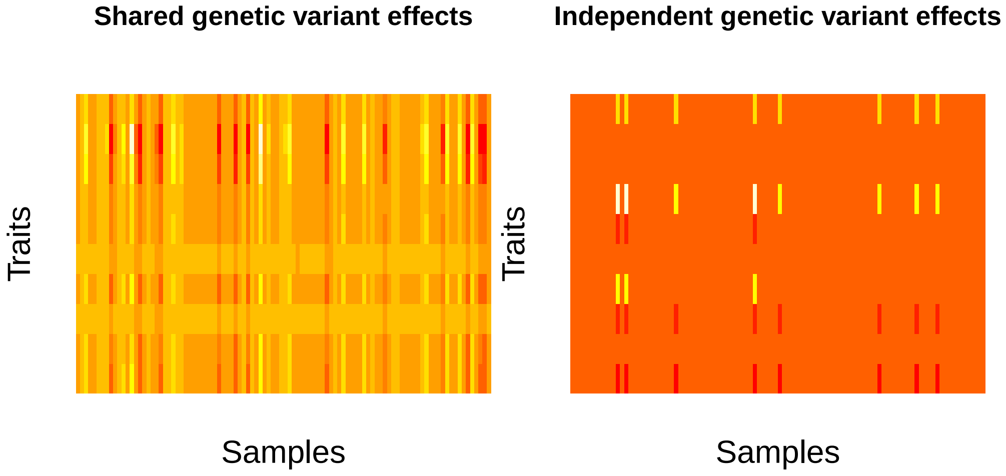

2. Infinitesimal genetic effects:

Infinitesimal genetic effects are simulated via `geneticBgEffects` based on the kinship estimates of the (simulated) samples. They can also be distinguished into a shared and independent effect. Both components are modeled by combining three matrix components : i) the kinship matrix K [N x N] which is treated as the sample-design matrix (the genetic profile of the samples), ii) a matrix B [N x P] with $vec(B)$ drawn from a normal distribution and iii) the trait design matrix A [P x P]. For the independent effect, A is a diagonal matrix with normally distributed values. A of the shared effect is a matrix of row rank one, with normally distributed entries in row 1 and zeros elsewhere. The three matrices are multiplied to obtain the desired final effect matrix E: $E = chol(K)BA$. As for the genetic variant effects, the kinship can either be estimated from simulated genotypes or read from file. The kinship is estimated as $K = XX_T$, with X the standardised genotypes of the samples. When estimating the kinship from the provided genotypes, the kinship is normalised by the mean of its diagonal elements and 1e-4 added to the diagonal for numerical stability. The provided kinship contains estimates for 50 samples across the entire genome.

```
## a) Estimate kinship from simulated genotypes
genotypes <- simulateGenotypes(N = 100, NrSNP = 10000,
                               frequencies = c(0.05, 0.1, 0.3,0.4),
                               verbose = FALSE)

genotypes_sd <- standardiseGenotypes(genotypes$genotypes)
kinship <- getKinship(N=100, X=genotypes_sd, verbose = FALSE)

## b) Read kinship from external kinship file
kinshipFile <- system.file("extdata/kinship/", "kinship.csv",
                           package = "PhenotypeSimulator")
kinshipFromFile <- getKinship(N=50, kinshipfile = kinshipFile,
                              verbose = FALSE)

genBg <- geneticBgEffects(N=100, kinship = kinship, P = 15)
```

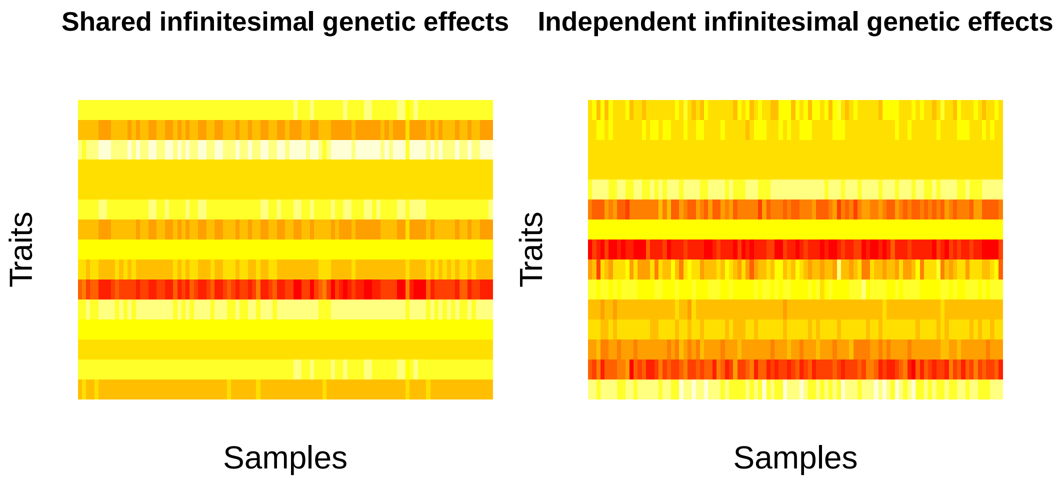

3. Non-genetic covariate effects:

Non-genetic covariate effects can be understood as confounding variables/covariates in an analysis, such as sex (binomial), age (normal/uniform), weight (normal) or disease status (categorical). Confounders can have effects across all traits (shared) or to a number of traits only (independent); the proportion of independent confounders from the total of simulated confounders can be chosen via `pIndependentConfounders`. The number of traits that are associated with the independent effects can be chosen via `pTraitIndependentConfounders`. Confounders can be simulated as categorical variables or following a binomial, uniform or normal distribution (as specified in `distConfounders`). Effect sizescan be simulated from a uniform or normal distribution, specified in `distBeta`. Multiple confounder sets drawn from different distributions/different parameters of the same distribution can be simulated by specifying `NrFixedEffects` and supplying the respective distribution parameters: i) `mConfounders` and `sdConfounders`: for the normal and uniform distributions, `mConfounders` is the mean/midpoint and `sdConfounders` the standard deviation/distance from midpoint, respectively; ii) `catConfounders` is the number of categorical variables to simulate; iii) `probConfounders` is the probability of success in the binomial distribution (with one trial) iv) `mBeta` and `sdBeta` are the mean/midpoint and standard deviation/distance from midpoint of the normally/uniformly distributed effect sizes.

```
# create 1 non-genetic covariate effect affecting 30% of all simulated traits. The effect
# follows a uniform distribution between 30 and 40  (resembling for instance age
# in a study cohort).
fixedNoiseUniform <- noiseFixedEffects(N = 100, P = 10, NrConfounders = 1,
                                       pIndependentConfounders = 1,
                                       pTraitIndependentConfounders = 0.3,
                                       distConfounders = "unif",
                                       mConfounders = 35, sdConfounders = 5)

# create 2 non-genetic covariate effects with 1 specific confounder affecting
# 20% of all simulated traits. The effects follow a normal distribution
fixedNoiseNormal <- noiseFixedEffects(N = 100, P = 10, NrConfounders = 2,
                                      pIndependentConfounders = 0.5,
                                      pTraitIndependentConfounders = 0.2,
                                      distConfounders = "norm",
                                      mConfounders = 0, sdConfounders = 1)

# create 1 non-genetic covariate effects affecting  all simulated traits. The
# effect follows a binomial distribution with probability 0.5 (resembling for
# instance sex in a study cohort).
fixedNoiseBinomial <- noiseFixedEffects(N = 100, P = 10, NrConfounders = 1,
```

```
                                               pIndependentConfounders = 0,
                                               distConfounders = "bin",
                                               probConfounders = 0.5)
```

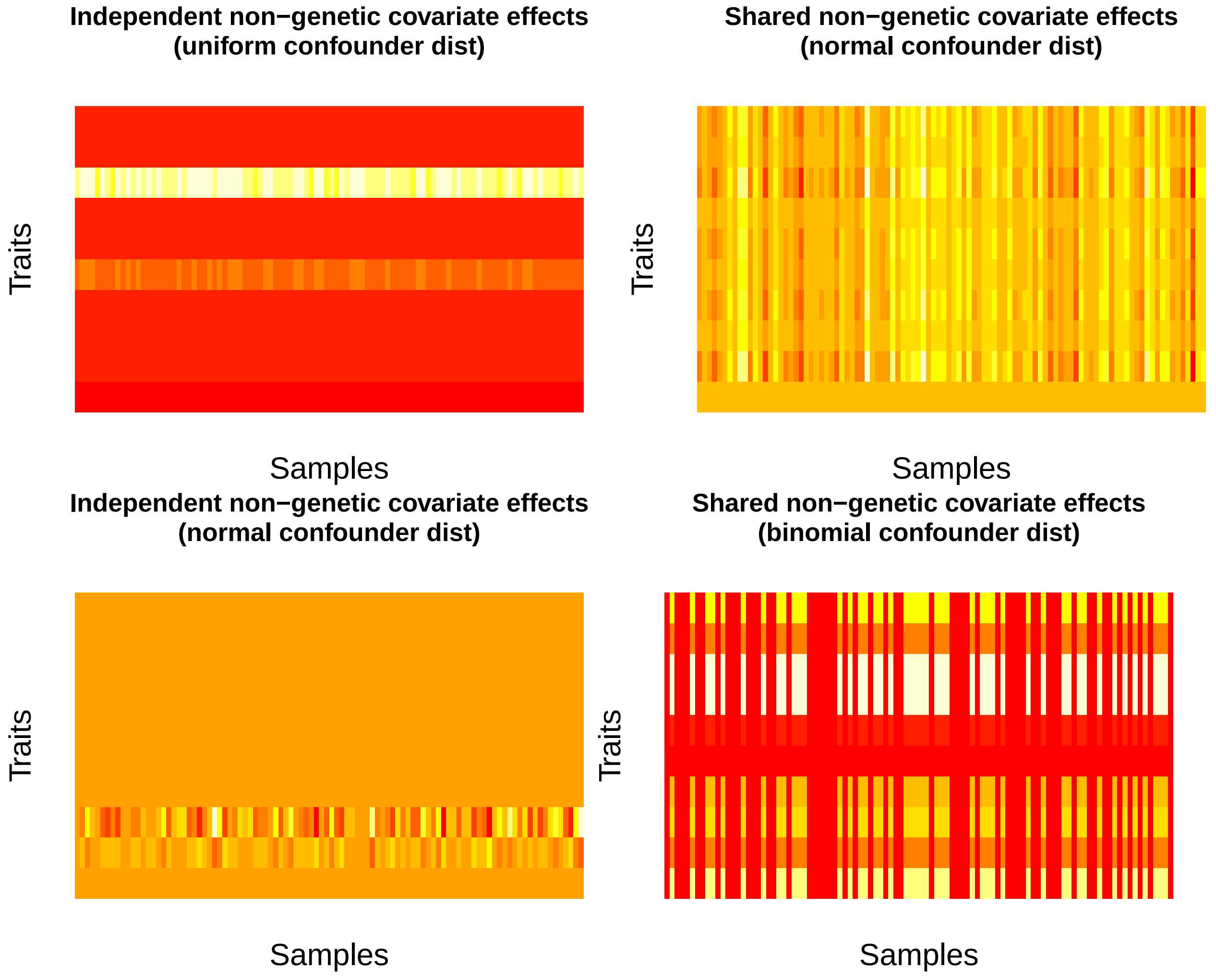

4. Correlated effects:

`correlatedBgEffects` can be used to simulate phenotypes with a defined level of correlation between traits. For instance, such effects can reflect correlation structure decreasing in phenotypes with a spatial component. The correlation matrix can either be supplied by the user or be constructed within *PhenotypeSimulator*. Here, the level of correlation depends on the distance of the traits. Traits of distance $d = 1$ (adjacent columns) will have correlation $cor = pcorr^1$, traits with $d = 2$ have $cor = pcorr^2$ up to traits with $d = (P-1)$ $cor = pcorr^{(P-1)}$. The correlated effect *correlated* is simulated as multivariate normal distributed with the described correlation structure C as the covariance between the phenotypic traits: *correlated* $N_{NP}(0, C)$.

```
# simulate correlated noise effect for 10 traits with top-level
# correlation of 0.8
correlatedNoise <- correlatedBgEffects(N = 100, P = 10, pcorr = 0.8 )

# correlation structure of the traits: strong the closer to the diagonal,
# little correlation at the furthest distance to the diagonal
furthestDistCorr <- 0.4^(10-1)
```

```
pairs(correlatedNoise$correlatedBg, pch = ".",
      main=paste("Correlation at furthest distance to diagonal:\n",
                 furthestDistCorr), cex.main=0.8)
image(correlatedNoise$correlatedBg, main="Correlated effects",  axes=FALSE,
      cex.main=0.8)
mtext(side = 1, text = "Samples", line = 1)
mtext(side = 2, text = "Traits", line = 1)
```

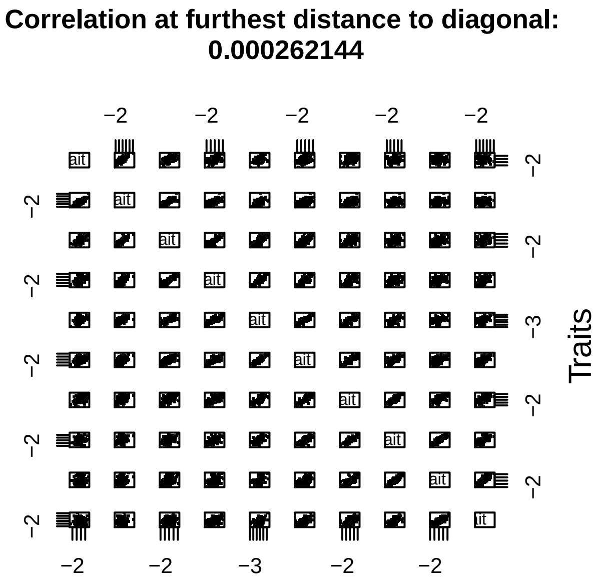

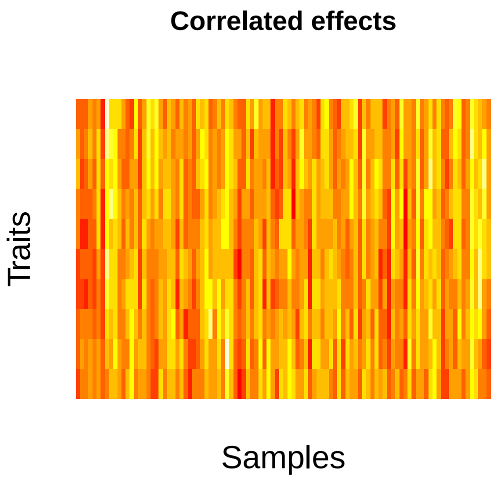

5. Observational noise effects:

Observational noise effects are simulated as the sum of a shared and independent effect. The independent effect is simulated as $vec(indpendent)\ N(mean, sd)$. The shared random effect is simulated as the matrix product of two normal distributions A [N x 1] and B [P x 1]: $shared = AB^T$

```
# simulate a noise random effect for 10 traits
noiseBg <- noiseBgEffects(N = 100, P = 10, mean = 0, sd = 1)
```

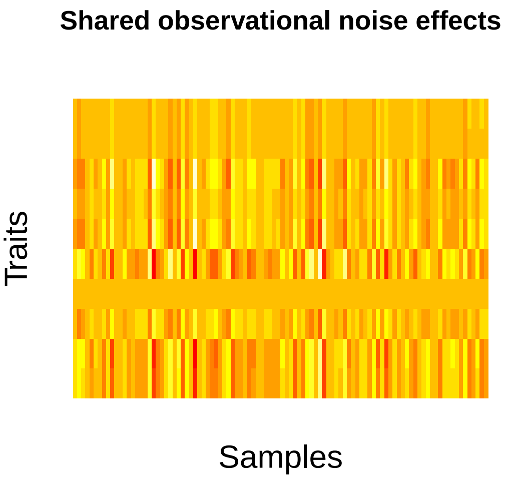

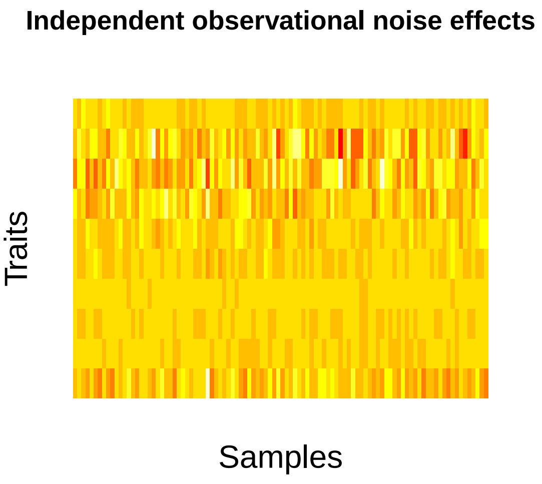

Supplement: Supplementary Data [file bty197_supp.zip › bty197-suppl_data/UsagePhenotypeSimulator.pdf]
